# Sub-Nanometer-Range Structural Effects From Mg^2+^ Incorporation in Na-Based Borosilicate Glasses Revealed by Heteronuclear NMR and MD Simulations

**DOI:** 10.1021/acs.jpcb.4c01840

**Published:** 2024-07-09

**Authors:** Peng Lv, Baltzar Stevensson, Renny Mathew, Tieshan Wang, Mattias Edén

**Affiliations:** † MOE Frontiers Science Center for Rare Isotopes, Lanzhou University, Lanzhou, Gansu 730000, PR China; ‡ Key Laboratory of Special Function Materials and Structure Design Ministry of Education, Lanzhou University, Lanzhou, Gansu 730000, PR China; § Physical Chemistry Division, Department of Materials and Environmental Chemistry, Arrhenius Laboratory, Stockholm University, SE-106 91 Stockholm, Sweden

## Abstract

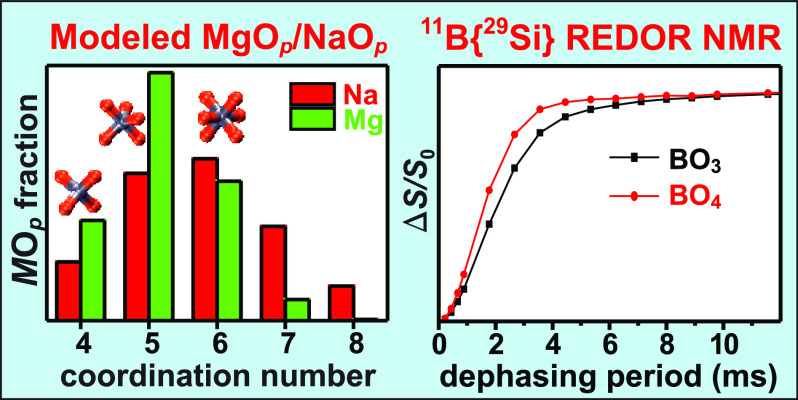

Magic-angle-spinning (MAS) nuclear magnetic resonance (NMR) experiments and molecular dynamics (MD) simulations were employed to investigate Na_2_O–B_2_O_3_–SiO_2_ and MgO–Na_2_O–B_2_O_3_–SiO_2_ glass structures up to ≈0.3 nm. This encompassed the {Na^[*p*]^}, {Mg^[*p*]^}, and {B^[3]^, B^[4]^} speciations and the {Si, B^[*p*]^, *M*^[*p*]^}–BO and {Si, B^[*p*]^, *M*^[*p*]^}–NBO interatomic distances to the bridging oxygen (BO) and nonbridging oxygen (NBO) species, where the superscript indicates the coordination number. The MD simulations revealed the dominance of Mg^[5]^ coordinations, as mirrored in average Mg^2+^ coordination numbers in the 5.2–5.5 range, which are slightly lower than those of the larger Na^+^ cation but with a narrower coordination distribution stemming from the higher cation field strength (CFS) of the smaller divalent Mg^2+^ ion. We particularly aimed to elucidate such Na^+^/Mg^2+^ CFS effects, which primarily govern the short-range structure but also the borosilicate (BS) glass network order, where both MD simulations and heteronuclear double-resonance ^11^B/^29^Si NMR experiments revealed a reduction of B^[4]^–O–Si linkages relative to B^[3]^–O–Si upon Mg^2+^-for-Na^+^ substitution. These effects were quantified and discussed in relation to previous literature on BS glasses, encompassing the implications for simplified structural models and descriptions thereof.

## 1 Introduction

Incorporating highly charged and/or small electropositive cations in oxide glasses, in particular rare-earth (RE^3+^) ions, often improves their thermal and mechanical properties.^1−8^ However, the high cost and toxicity of RE^3+^ species make Mg^2+^ an inexpensive and environmental-friendly alternative for glasses in a sustainable future society, as the *cation field strength* (CFS) of the small Mg^2+^ cation is only marginally lower than that of La^3+^. (The CFS scales as the charge divided by the square of the cation radius).^9^ There has been a decades-long and recently growing interest in exploring the structures of Mg^2+^-bearing aluminosilicate (AS) glasses^10−15^ and their correlation with physical properties.^16−19^ In contrast, there are comparatively few studies of incorporating Mg^2+^ into the other main glass family of ubiquitous technological and geological importance, i.e., *borosilicate* (BS) glasses.^19−23^ It also remains unclear as to whether the physical-property boosts observed for Mg^2+^-bearing AS glasses (relative to those with lower-CFS alkali/alkaline earth metal ions) also apply to the BS glass context. Results from *M*O–Na_2_O–B_2_O_3_–SiO_2_ glasses merely suggest that the larger Sr^2+^ and Ba^2+^ cations offer higher hardness and better elastic properties than their Mg^2+^ counterpart.^22^

These differences may be traced to the distinct effects from high-CFS Mg^2+^ and RE^3+^ incorporation in AS versus BS structures. While the physical glass properties are enhanced slightly from stronger Mg^2+^/RE^3+^–O bonds relative to those of lower-CFS *M*^+^/*M*^2+^ glass-network modifiers, all *M*–O bonds remain markedly weaker than their *F*–O counterparts, where *F* denotes a *network forming* species, *F* = {Si, Al, B}. Rather, the structure-strengthening effects and accompanying improved thermal/mechanical properties upon high-CFS *M*^*z*+^ inclusion in AS glasses stem from the effects on the Al speciation, where the dominant AlO_4_ groups partially convert into higher-coordination yet network-forming AlO_5_ and AlO_6_ polyhedra,^24,25^ whose higher network cross-linking enhances the physical glass properties.^5,7,25,26^ Including high-CFS *M*^*z*+^ cations in BS glasses, on the other hand, strongly alters their B speciations. B-bearing glass networks comprise both BO_3_ (B^[3]^ coordinations) and BO_4_ (B^[4]^) groups,^24,27^ where large B^[4]^ populations increase the network connectivity and boost many glass properties, such as the hardness and the glass transition temperature.^22,26,28−31^ However, high-CFS cations tend to promote B^[3]^ formation at the expense of B^[4]^,^19,22,32−37^ which lowers the overall glass-network connectivity and may thereby degrade physical properties. Yet, little is known about the precise structure–property relationships of high-CFS *M*^*z*+^-bearing BS glasses, which are strongly dependent on the precise glass stoichiometry and likely also on the *M*^*z*+^ size (alone);^22^ for example, incorporating Mg^2+^ into a few Na_2_O–B_2_O_3_–SiO_2_ glass compositions reduced their hardness and elastic properties, whereas these properties improved when instead introducing the larger high-CFS La^3+^ cation.^22^

By utilizing atomistic molecular dynamics (MD) simulations and magic-angle-spinning (MAS) nuclear magnetic resonance (NMR) spectroscopy, we report here on the structural alterations from Mg^2+^-for-Na^+^ substitutions in four Na_2_O–B_2_O_3_–SiO_2_ base-glass compositions. Besides the {B^[*p*]^} and {O^[*q*]^} speciations, we examine the local Na and Mg coordination environments and their distinctly different propensities for coordinating the bridging oxygen (BO; O^[2]^ coordinations) and nonbridging oxygen (NBO; O^[1]^) species. We particularly aim to understand the dependence of the local glass structure on the *M*^*z*+^ CFS, which besides Na^+^ and Mg^2+^ also involves results from Ca^2+^-bearing BS glass models along with previously published data from RE^3+^ cations in AS glasses.

We then move the spotlight from the first *F*^[*p*]^ and *M*^[*p*]^ coordination shells onto the *F*–O–*F*′ glass-network linkages, where the silicate and borate group intermixing was probed by computational modeling and heteronuclear double-resonance ^11^B/^29^Si MAS NMR experiments. Current B/Si interconnectivity insights partially stem from ^17^O triple-quantum MAS (3QMAS)^38^ experimentation.^24,36,37,39,40^ Although widely applied, it may not unambiguously discriminate between the B^[3]^ and B^[4]^ coordination numbers of the B–O–Si and B–O–B linkages (let alone *quantify* them, although such claims have been made^36,37,40^). The heteronuclear magnetic ^11^B–^29^Si dipolar interaction, which is mediated directly through space and scales as the inverse cube of the ^11^B–^29^Si distance,^24,25,41,42^ offers a more direct probing of the ^11^B^[*p*]^/^29^Si intermixing in BS glasses. However, its application is relatively sparse,^43−49^ mainly stemming from the requirement of dedicated glass syntheses from costly ^29^Si-enriched silica to enable high-quality experimental data for quantitative analyses, which is otherwise severely hampered or even precluded by the low natural abundance (4.7%) of the NMR-active ^29^Si isotope. The impact of Mg^2+^ incorporation on the relative degrees of B^[3]^–O–Si and B^[4]^–O–Si bonding in the Mg/Na-bearing BS glass networks is discussed in relation to current literature, as well as the implications for existing simplified BS-glass structure descriptions/models.

## 2 Materials and Methods

### 2.1 Borosilicate Glasses

Our study involved the eight BS glass compositions listed in Table 1, encompassing four ternary *R*Na_2_O–B_2_O_3_–*K*SiO_2_ glasses and four quaternary *R*[0.5MgO–0.5Na_2_O]–B_2_O_3_–*K*SiO_2_ analogs. They constitute a subset of a large BS glass ensemble examined previously.^22,50,51^ We adopt the glass nomenclature of ref (50), where each Na- and Mg/Na-based glass is denoted by Na*K*–*R* and MgNa*K*–*R*, respectively, with the {*K*, *R*} parameters defined by^52,53^
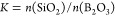
1
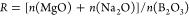
2Each nominal glass composition in Table 1 is expressed in terms of its oxide equivalents and the atomic fraction of each element *E* in the (Mg)–Na–B–Si–O glass,
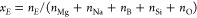
3where *n*_*E*_ is the corresponding stoichiometric amount.

**Table 1:** Borosilicate Glass Compositions^a^

	oxide equivalents (mol %)				atomic fractions					BO_4_ fractions^b^		NBO fractions^c^	
glass	MgO	Na_2_O	B_2_O_3_	SiO_2_	*x*_Mg_	*x*_Na_	*x*_B_	*x*_Si_	*x*_O_	NMR	MD	NMR	MD
***R* = 0.75**													
Na2.0–0.75		20.0	26.7	53.3		0.114	0.151	0.151	0.584	0.604	0.547	0.038	0.054
MgNa2.0–0.75	10.0	10.0	26.7	53.3	0.029	0.058	0.155	0.155	0.603	0.361	0.353	0.100	0.104
Na4.0–0.75^d^		13.0	17.4	69.6		0.078	0.104	0.208	0.610	0.623; **0.618**	0.527	0.022; **0.022**	0.039
MgNa4.0–0.75^d^	6.5	6.5	17.4	69.6	0.020	0.040	0.106	0.212	0.622	0.313; **0.310**	0.301	0.074; **0.074**	0.077

***R* = 2.1**													
Na2.0–2.1		41.2	19.6	39.2		0.242	0.116	0.116	0.526	0.487	0.447	0.354	0.364
MgNa2.0–2.1	20.6	20.6	19.6	39.2	0.065	0.129	0.123	0.123	0.560	0.402	0.364	0.373	0.382
Na4.0–2.1		29.6	14.1	56.3		0.180	0.086	0.172	0.562	0.697	0.608	0.214	0.228
MgNa4.0–2.1^d^	14.8	14.8	14.1	56.3	0.094	0.047	0.090	0.180	0.589	0.459;**0.445**	0.444	0.249;**0.251**	0.253

a Nominal ternary *R*Na_2_O–B_2_O_3_–*K*SiO_2_ (“Na*K*–*R*”) or quaternary *R*[0.5MgO–0.5Na_2_O]–B_2_O_3_–*K*SiO_2_ (“MgNa*K*–*R*”) glass compositions and their corresponding atomic fractions {*x*_Mg_, *x*_Na_, *x*_B_, *x*_Si_, *x*_O_} defined by *x*_*E*_ = *n*_*E*_/(*n*_Mg_ + *n*_Na_ + *n*_B_ + *n*_Si_ + *n*_O_).

b Fractional populations of B^[4]^ coordinations, , as obtained either by ^11^B NMR or by MD simulations, with the respective uncertainties of ±0.01 and ±0.005. The experimental  values listed to the left are reproduced from Lv et al.,^22^ while those to the right (in bold) are results from the present ^29^Si-enriched glasses; the latter data are employed throughout the experimental analyses of those glass specimens.

c NBO fraction, *x*_NBO_, out of all BO and NBO species, as either obtained by MD simulations (uncertainty ±0.001) or calculated from eq 9 (±0.01) by using the NMR-derived {} data. The experimental *x*_NBO_ values listed to the left and right (in bold) correspond to the results presented by Lv et al.^22^ and those of the present ^29^Si-enriched glasses, respectively; the latter data are assumed throughout this work.

d Prepared with ^29^Si isotopic enrichment (section 2.2).

MD simulations were performed for all glass compositions in Table 1. The need for dedicated glasses synthesized from costly ^29^SiO_2_ to enable the heteronuclear ^11^B/^29^Si NMR experiments, however, limited them to three specimens: Na4.0–0.75, MgNa4.0–0.75, and MgNa4.0–2.1.

The CFS of an *M*^*z*+^ cation is defined according to^9^
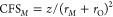
4where *r*_*M*_ is the cation radius and *r*_O_ = 1.36 Å. Table S1 lists the CFS values of the *F* = {Si, B^[3]^, B^[4]^} network formers and the *M*^*z*+^ = {Na^+^, Mg^2+^} network modifiers primarily targeted here, along with a few other *M*^*z*+^ cations discussed in section 3. For consistency, we employed *r*_*M*_ values for sixfold-coordinated *M*^*z*+^ species (*M*^[6]^) throughout. The coordination number is not indicated for exclusively tetrahedrally coordinated Si ≡ Si^[4]^ atoms.

### 2.2 Glass Preparation

The ^29^Si-enriched Na4.0–0.75, MgNa4.0–0.75, and MgNa4.0–2.1 glasses were prepared in 250 mg batches from analytical grade ^29^SiO_2_ (99.8% ^29^Si), H_3_BO_3_, Na_2_CO_3_, and MgO precursors. After removing potential OH/H_2_O contaminations by preheating the ^29^SiO_2_ powder at 950 °C for 24 h, the precursors were mixed thoroughly in a mortar, transferred to a Pt crucible, and decarbonated at 950 °C for 2 h before being heated to final melt temperatures of 1000, 1100, and 1200 °C for the MgNa4.0–2.1, Na4.0–0.75, and MgNa4.0–0.75 batches, respectively. Those temperatures were deduced from multiple preparations using regular (nonenriched) SiO_2_ so as to ensure complete melting but minimal evaporation losses and close fictive temperatures to previous BS glass specimens of identical nominal compositions but prepared in larger batches (6 g) at ≈200 °C higher melting temperatures.^22^ The melt was held for 20 min and then quenched by immersing the crucible bottom in cold water.

All glass specimens were free of crystalline impurities. Their compositions are expected to be close to their batched/nominal counterparts listed in Table 1, as corroborated by the minute evaporation losses during heating of 1.0 wt % and 1.6 wt % for MgNa4.0–2.1 and MgNa4.0–0.75, respectively. Although the Na4.0–0.75 glass revealed a markedly higher loss (5.6% wt %) than the 1–2 wt % we normally observe,^22,34^ it mainly reflects accidental melt-loss prior to quenching. Indeed, relative to the batched B_2_O_3_ contents, the B_2_O_3_ masses determined from ^11^B MAS NMR experiments calibrated to H_3_BO_3_ as the standard (see refs (22) amd (34)) revealed marginal relative deviations from 0.6% for Na4.0–0.75 to 1.7% for MgNa4.0–0.75.

### 2.3 Molecular Dynamics Simulations

Atomistic MD simulations mimicking a melt-quench process were utilized to produce BS glass models with the stoichiometries from Table 1. The computations utilized the DLPOLY4.09 program,^54^ where *NVT* ensembles in a cubic box with periodic boundary conditions were simulated using a box size and number of atoms (6600–11600) to match the nominal chemical glass composition and the experimental density (Table S2). Each melt-quench protocol started from randomly positioned atoms, which were equilibrated for 100 ps at 3500 K, followed by a stepwise temperature reduction (5 K/ps) to 300 K. The equations of motion were integrated in steps of 0.2 fs by using the velocity Verlet integrator, while the temperature was controlled by a gentle stochastic thermostat with a 1.0 ps time constant and a 1.0 ps^–1^ Langevin friction constant. The structural data were sampled and averaged over the last 150 ps of a final 200 ps equilibration stage. The average value and uncertainty of each reported structural parameter were obtained by performing the melt-quench protocol four times. The selected system sizes, equilibration stages, and other simulation conditions provide well-converged and reliable structural parameters.^34,55−57^

All simulations utilized a polarizable shell-model potential.^58−60^ Every cation carries its full formal charge,^58^ but the O^2–^ species are represented by core (O_C_) and shell (O_S_) portions with masses *m*_C_ = 15.7994 u and *m*_S_ = 0.2000 u, respectively, and corresponding charges *z*_C_ = +0.8482*e* and *z*_S_ = −2.8482*e* (obeying *z*_C_ + *z*_S_ = −2), where “u” is the atomic mass unit and *e* is the elementary charge. Each core–shell unit is connected by a harmonic potential with a force constant of 74.92 eV/Å ^2^.^58^ The interaction energy of two atom/ion species α and β separated by a distance *r*_αβ_ was modeled by a modified Buckingham potential
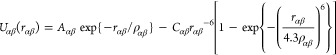
5that accounted for all short-range O_S_–O_S_ and cation–O_S_ pair interactions. It was evaluated out to *r*_αβ_ = 0.8 nm. The B–O force field conforms to eq 5 with *C*_αβ_ ≡ 0 but also includes a repulsive  term.^55,56^Table S3 compiles all  parameters, encompassing a three-atom potential for constraining the O–Si–O intratetrahedral angles. Long-range Coulombic interactions were calculated by a smoothed particle-mesh Ewald summation^54^ with a 1.2 nm real-space cutoff and an accuracy of 10^–6^.

### 2.4 Solid-State NMR Experiments

All NMR experiments were performed with Bruker Avance-III spectrometers. The ^29^Si MAS NMR spectra were acquired at a magnetic field (*B*_0_) of 9.4 T (79.47 MHz ^29^Si Larmor frequency) using 4 mm zirconia rotors undergoing MAS at ν_*r*_ = 14.00 kHz and radio frequency (rf) pulses with a ≈70° flip angle (ν_Si_ = 84 kHz nutation frequency), relaxation delays of 3600 s, and 16 accumulated NMR signal transients. The ^11^B (spin-3/2) NMR spectra were recorded at *B*_0_ = 14.1 T (−192.5 MHz ^11^B Larmor frequency) and ν_*r*_ = 24.00 kHz using full 3.2 mm zirconia rotors and strong/short rf pulses (0.33 μs, 13° flip angle, ν_B_ = 105 kHz). The {B^[3]^, B^[4]^} populations of each glass were determined from the integrated central-transition (CT) NMR-signal intensities,^22,50^ which were corrected for the satellite-transition centerband peak that overlaps with the main CT ^11^B^[4]^ signal by using standard procedures.^61^ Every ^11^B NMR spectrum was corrected for probehead “background” signals by subtracting the result from the empty rotor recorded under otherwise identical experimental conditions. ^29^Si (δ_Si_) and ^11^B (δ_B_) shifts were referenced relative to neat tetramethylsilane (TMS) and BF_3_·OEt_2_, respectively.

The Van Vleck *dipolar second moment*(62) is proportional to the sum over the inverse sixth power of the interatomic distance of each heteronuclear S_*j*_–I_*k*_ spin-pair in a structure: 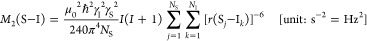
6Here, μ_0_ is the permeability of vacuum, γ_I_ (γ_S_) is the magnetogyric ratio of spin species I (S), and *N*_I_ and *N*_S_ are the respective total numbers of I and S nuclei in the glass obtained from its stoichiometry with , where *N*_A_ is Avogadro’s number and  the natural isotopic abundance. *I* denotes the spin quantum number of the nuclide I, i.e., *I* = 1/2 (^29^Si) and *I* = 3/2 (^11^B) for the respective *M*_2_(B^[*p*]^–Si) and *M*_2_(Si–B^[*p*]^) entities. We stress the unit of s^–2^ in eq 6, which is consistent with our previous work^57,63,64^ but differs from the original *M*_2_ definition with units of rad^2^/s^2^,^62^ which is most frequently encountered in the literature (e.g., refs (19), (41), and (42)). The two dipolar second-moment definitions are related by 4π^2^*M*_2_[s^–2^] = *M*_2_[rad^2^/s^2^]. Units aside, we comment that incorrect *M*_2_(S–I) expressions are stated in two recent review articles,^24,25^ which should appear as in ref (64) and eq 6.

The *M*_2_(B^[*p*]^–Si) value of a given BS glass may be estimated from a ^11^B{^29^Si} REDOR NMR experiment^65^ that restores/“recouples” the MAS-averaged ^11^B^[*p*]^–^29^Si dipolar-interaction effects during a recoupling period (τ_rec_) by a series of rotor-synchronized 180° rf pulses applied to ^29^Si, during which the ^11^B^[*p*]^ NMR signal [*S*(τ_rec_)] becomes attenuated (“dephased”) relative to that obtained by a spin–echo experiment [*S*_0_(τ_rec_)]^65^ obtained by CT-selective ^11^B rf pulses.

All double-resonance ^11^B{^29^Si} REDOR NMR experiments were performed at *B*_0_ = 14.1 T and ν_*r*_ = 9.00 kHz with each glass powder centered to the 1/3 volume of a 4 mm zirconia rotor to reduce the impact from rf inhomogeneity.^41,66,67^ All experiments were started by a saturation-recovery rf-pulse comb followed by a 1.5 s relaxation delay. The 180° dipolar recoupling pulses operated at ν_Si_ = 46 kHz with the XY8 phase-cycling scheme to minimize rf-pulse errors.^68^ The ^11^B 90° and 180° spin–echo rf pulses were 17.0 μs and 34.0 μs, respectively. The dipolar recoupling period was sampled out to several ms at even integer multiples *n* of the rotor period, . Each *M*_2_(B^[3]^–Si) and *M*_2_(B^[4]^–Si) value was extracted from the respective integrated ^11^B^[3]^ and ^11^B^[4]^ NMR intensities of the *S*_0_(τ_rec_) and *S*(τ_rec_) spectra, whereupon the resulting {τ_rec_, Δ*S*/*S*_0_} data (restricted to Δ*S*/*S*_0_ ⩽ 0.2^24,41,42^) were fitted to the expression^41,42,63^
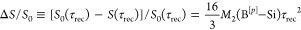
72–4 independent NMR-data blocks with 256–512 accumulated signal transients per block were acquired for each glass specimen. The average value of each *M*_2_(B^[*p*]^–Si) estimate and its uncertainty were extracted from these independent {*M*_2_(B^[*p*]^–Si)} best-fit results.

## 3 Results and Discussion

### 3.1 Boron and Oxygen Speciations

**Figure 1:**
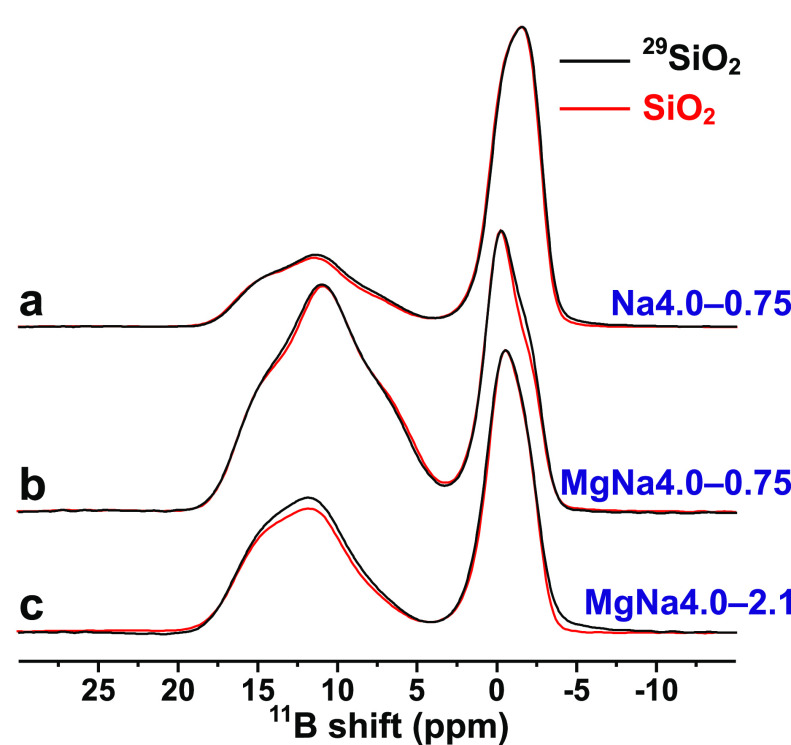
^11^B MAS NMR spectra recorded at 14.1 T and 24.00 kHz MAS from (a) Na4.0–0.75, (b) MgNa4.0–0.75, and (c) MgNa4.0–2.1 glass specimens prepared from ^29^SiO_2_ (black traces) or from SiO_2_ with ^29^Si at natural abundance (red traces; reproduced from Lv et al.^22^). The glasses were prepared under similar conditions and reveal very similar ^11^B NMR spectra and borate speciations (Table 1).

All BS glasses considered herein comprise networks of interconnected SiO_4_, BO_3_ and [BO_4_]^−^ groups, where  and  denote the respective fractional populations of B^[3]^ and B^[4]^. Table 1 lists each  value obtained from either the ^11^B MAS NMR spectrum or MD simulations, whereas the corresponding BO_3_ fraction is given from the following normalization:
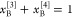
8The experimental {} data were reproduced from ref (22) except for the three ^29^Si-enriched Na4.0–0.75, MgNa4.0–0.75, and MgNa4.0–2.1 specimens that were prepared specifically for the present study. Their borate speciations match very well those of glasses prepared from regular (non-^29^Si-enriched) SiO_2_,^22^ suggesting very close stoichiometries and fictive temperatures for both specimens of each Na4.0–0.75, MgNa4.0–0.75, and MgNa4.0–2.1 stoichiometry (see Table 1 and Figure 1). The MD simulations revealed O speciations solely comprising NBO and BO sites, whereas “free O^2–^ anions” (O^[0]^) are absent throughout and the “O tricluster” (O^[3]^) populations remain <0.02% out of each {O^[*q*]^} ensemble. Hence, the fractional populations of NBO (*x*_NBO_) and BO (*x*_BO_) species obey *x*_NBO_ + *x*_BO_ = 1.

The Na^+^/Mg^2+^ cations compensate the negative charges of the [BO_4_]^−^ and NBO (O^–^) moieties, implying that the fractional populations of B^[4]^ and NBO anions are coupled according to . Hence, *x*_NBO_ is readily deduced from the glass stoichiometry and the ^11^B NMR-derived  value as follows:
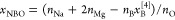
9Equation 9 reflects the well-known dual “network modifier” and “charge compensator” structural properties of the *M*^*z*+^ cations in borate-based glasses,^24,27,52,53,69,70^ which result in *M*^*z*+^···O^–^ and *M*^*z*+^···[BO_4_]^−^ structural motifs from the respective BO → NBO and B^[3]^ → B^[4]^ conversions. The borate speciation of a BS-based glass depends strongly on both its stoichiometry and CFS_*M*_.^19,22,32−37^ As expected from the higher CFS_Mg_ = 0.46 Å^–2^ than CFS_Na_ = 0.18 Å^–2^,^19,22,32,33^ introducing Mg^2+^ to a Na*K*–*R* glass boosts the NBO content for fixed {*K*, *R*} parameters, while the B^[4]^ population is markedly reduced (Table 1). This effect is particularly drastic for the Na*K*–0.75 and Na4.0–2.1 glasses with : when half of the Na^+^ ensemble is replaced by Mg^2+^, a significant B^[4]^ → B^[3]^ conversion occurs, rendering BO_3_ groups most abundant in all MgNa*K*–*R* specimens.

For a clear-cut discrimination between the *M*^*z*+^ and *F* species, we employ the ancient “network modifier” terminology when referring to the *M*^+^/*M*^2+^ cations, although the categorical “modifier” and “charge-compensator” classification is oversimplified.^56,57^ For instance, both MD simulations and NMR experiments reveal that a significant/major portion of the {Na^+^} ensemble associates with the *formally* charge-neutral Si(O^[2]^)_4_ and B(O^[2]^)_3_ moieties via Na^+^···O–Si/B^[3]^ fragments (rather than with the negatively charged [BO_4_]^−^ and *F*–NBO moieties), rendering Na–BO contacts prevalent for all but very NBO-rich glasses.^56,57^

A long-standing problem of classical MD simulations of B-bearing glasses is their (in)accurate {, } predictions, which not only depend strongly on the precise glass composition but also on thermal history of the glass due to the temperature-dependent BO_3_ ⇄ BO_4_ equilibrium.^71−73^ The engineering of reliable B–O force fields has received significant recent attention.^74−81^ The modeled BO_4_ and NBO populations listed in Table 1 were obtained with the B–O interatomic potential parameters of refs (55) and (56), which have been validated for Na- and Na/Ca-bearing borate and boro(phospho)silicate glasses over large composition domains.^34,55,56,82^ Contrasting the modeled and experimental BO_4_ populations of Table 1, however, reveals a highly variable performance. The agreement is excellent for all MgNa*K*–*R* glasses (with *relative* deviations within 3%) but MgNa2.0–2.1, which along with all Na*K*–*R* models reveal markedly larger discrepancies to experiments, typically by ≈10% but with a substantial deviation of 17% for Na4.0–0.75. These errors stem from the distinctly different  ranges between the Na*K*–*R* and MgNa*K*–*R* glasses. As noted in refs (34), (55), and (56) but more clearly shown by Pedone and co-workers,^82,83^ MD simulations with our B–O force field are prone to underestimating the BO_4_ population: although the deviations are insignificant for glasses with , they grow progressively for increasing values of  (Table 1).

The underestimated (overestimated) modeled BO_4_ (BO_3_) populations are reasons for concern regarding reliable predictions of some *medium-range* (0.3–1 nm) glass-structure features. Gratifying, however, is the typically (very) good agreement observed *consistently* relative to experimental data on several interatomic-distance-related structural parameters, such as the relative B^[3]^/B^[4]^···Na^+^ and P–O–B^[*p*]^ contacts in boro(phospho)silicate glasses,^55,57^ as well as their B^[*p*]^–O–B^[*q*]^ linkage statistics,^34,50,55^ which constitute the *most sensitive* medium-range structural parameters on the precise {, } fractions; notwithstanding that the NMR/MD-derived B^[*p*]^–O–B^[*q*]^ populations do differ, *all* experimental findings were reproduced qualitatively/semiquantitatively by the glass models.^34,50,55^ Moreover, the relative B^[3]^/B^[4]^–O–Si contacts predicted by the glass models in section 3.5 agree very well with our experiments, *encompassing* the Na4.0–0.75 glass. Indeed, out of the plethora of B–O force fields proposed to date (e.g., refs (74−81)), *solely* the herein utilized option^55,56^ has been assessed extensively against experimental interatomic-distance-related parameters directly reflecting the *medium-range* glass organization.

### 3.2 MD-Derived Average *F*–O and *M*–O Distances

Here and in sections 3.3 and 3.4, we examine the MD-derived first coordination shells of the network formers and modifiers, paying particular attention to the structural bearings from Mg^2+^, thereby complementing our previous structural reports on large sets of Na^+^ and Na^+^/Ca^2+^ bearing borate and boro(phospho)silicate glasses with variable B, Si, and NBO contents.^56,57^

For each {Si, B^[3]^, B^[4]^} network former, Table 2 compiles the *average F*^[*p*]^–O^[*q*]^ interatomic distance, (*F*^[*p*]^–O^[*q*]^), for each of O^[1]^ and O^[2]^, along with the corresponding {(*F*^[*p*]^–O)} result that represents the weighted average of the *F*^[*p*]^–O^[1]^/O^[2]^ distances over all {*F*O_*p*_} polyhedra. We stress that each *F*^[*p*]^–O/O^[*q*]^ and *M*^[*p*]^–O/O^[*q*]^ interatomic distance (“bond-length”) discussed herein constitutes the arithmetic average across the entire *F*–O^[*q*]^ (*M*–O^[*q*]^) bond ensemble in the structure.  was *not* extracted from the respective pair-distribution maximum, which constitutes the *most probable F*–O distance but is frequently reported as (*F*–O) (e.g., see refs (60), (76), and (83−86)). Because the various Si/B^[*p*]^–BO/NBO distances are governed by the high-CFS Si^4+^ and B^3+^ cations (Table S1), they remain similar for all BS glasses regardless of the precise network modifier species. All *F*^[*p*]^–O^[*q*]^ distances in Table 2 conform well with those discussed by Stevensson et al.^56^ for Na–(Ca)–B–Si–O glasses, which agreed well with the sparsely available experimental data. The direct dependence of each *F*–O distance on the NBO content of the glass is evident when contrasting the (Si–O) and (B^[3]^–O) data from each Na*K*–*R* glass with its MgNa*K*–*R* analog (in contrast with (B^[4]^–O); vide infra): the distances in the Mg-bearing glass are marginally but consistently shorter by ≈0.3 pm due to the slightly higher NBO contents in those glasses coupled with the shorter *F*–NBO distances relative to *F*–BO (Table 2).

**Table 2:** MD-Derived Average *F*/*M*–O Bond Lengths^a^

		(B^[3]^–O^[*q*]^) (pm)			(B^[4]^–O^[*q*]^) (pm)			(Si–O^[*q*]^) (pm)			(Na–O^[*q*]^) (pm)			(Mg–O^[*q*]^) (pm)		
glass	*x*_NBO_	O	O^[2]^	O^[1]^	O	O^[2]^	O^[1]^	O	O^[2]^	O^[1]^	O	O^[2]^	O^[1]^	O	O^[2]^	O^[1]^
***R* = 0.75**																
Na2.0–0.75	0.054	135.33	135.45	134.00	142.53	142.59	134.69	163.96	164.03	160.29	257.9	260.8	238.4			
MgNa2.0–0.75	0.104	135.10	135.29	133.85	143.07	143.31	134.84	163.65	163.73	160.96	258.6	261.0	245.5	213.0	225.8	203.2
Na4.0–0.75	0.039	134.90	134.97	133.88	142.09	142.13	134.71	163.58	163.64	160.09	258.5	261.7	236.4			
MgNa4.0–0.75	0.077	134.72	134.83	133.72	142.78	142.99	134.93	163.33	163.40	160.71	258.9	261.9	244.0	211.4	227.7	201.6

***R* = 2.1**																
Na2.0–2.1	0.364	136.72	138.37	134.57	144.20	145.43	134.83	164.69	165.46	161.26	252.4	262.5	243.7			
MgNa2.0–2.1	0.382	136.26	137.56	134.54	144.60	146.17	135.21	164.49	165.09	161.69	255.9	262.5	248.6	210.3	228.3	206.0
Na4.0–2.1	0.228	136.25	137.20	134.27	143.08	143.68	134.79	164.30	164.78	160.73	254.0	263.1	241.3			
MgNa4.0–2.1	0.253	135.85	136.63	134.25	143.66	144.59	135.13	164.02	164.37	161.31	257.3	263.4	246.9	209.6	228.7	204.3
σ^b^	0.001	0.02	0.03	0.02	0.04	0.05	0.08	0.02	0.02	0.04	0.1	0.1	0.2	0.3	0.4	0.1

a Average *F*^[*p*]^–O^[*q*]^ and *M*–O^[*q*]^ distances between the NBO (O^[1]^ coordination) and BO (O^[2]^) species for *F* = {Si^[4]^, B^[3]^, B^[4]^} and *M* = {Na, Mg}, where the latter involve the entire {Na^[*p*]^} and {Mg^[*p*]^} ensembles. The (B^[*p*]^–O), (Si–O), and (*M*–O) values constitute averages across all {O^[1]^, O^[2]^} sites.

b The data uncertainties are ±1σ, with σ given for each entity.

We next consider the *F*–O^[1]^ and *F*–O^[2]^ bond-length variations. The large CFS of the very small B^3+^ cation manifests as tightly confined B^[3]^–O^[1]^ and B^[4]^–O^[1]^ bond lengths (134–135 pm throughout), which are markedly shorter than those of Si–O^[1]^ (161–162 pm; see Table 2). Nonetheless, whereas the average B^[3]^–O^[2]^ distances are only a few pm longer than those of B^[3]^–O^[1]^, the B^[4]^–O^[2]^ bond lengths are 7–8 pm longer (142–146 pm) than their B^[4]^–O^[1]^ counterparts, which is consistent with previous findings.^75−77,84^Table 2 confirms the anticipated feature of nearly constant (*F*^[*p*]^–O^[1]^) values regardless of the *n*_Si_/*n*_B_ molar ratio of the glass (related to *K*) or its NBO content (related to *R*).^56^ In contrast, all {(*F*–O^[2]^)} values increase slightly for increasing *x*_NBO_. For instance, contrast the bond lengths from each Na*K*–0.75 and MgNa*K*–0.75 structure with those of its NBO-richer Na*K*–2.1 and MgNa*K*–2.1 counterpart: (*F*^[*p*]^–O^[2]^) is increased by 1–2 pm for Si and slightly more for the two B^[3]^ and B^[4]^ coordinations (2–3 pm; Table 2). However, for the Mg^2+^/NBO-richer MgNa*K*–2.1 glasses, for which any bond-length effect from Mg^2+^ is expected to be most pronounced, it is notable that (B^[3]^–O^[2]^) is *shorter* by ≈1 pm than that for their Na*K*–2.1 analogs, whereas the reverse trend of a ≈1 pm *longer*(B^[4]^–O^[2]^) bond length is observed.

We onward focus on the average *M*–O^[1]^, *M*–O^[2]^, and *M*–O distances listed in Table 2 for the Na^+^ and Mg^2+^ species, which are averages over all *M*^[*p*]^ coordinations in the structure. As expected, the Na/Mg–O^[1]^ bond lengths are significantly shorter than their Na/Mg–O^[2]^ counterparts. Marginal variations of the average Mg–O^[1]^ and Mg–O^[2]^ distances are observed throughout, regardless of the precise B, Si, or NBO content of the glass (Table 2). The mean Na–{O, O^[1]^, O^[2]^} bond lengths predicted from the Na*K*–*R* glass models conform well to those discussed for Na- and Na/Ca-bearing borate/BS glasses in ref (56). Notwithstanding that (Na–O^[2]^) only varies marginally among the eight glass structures (Table 2), the Na–O^[1]^ distances are markedly longer (by 5–8 pm) in each MgNa*K*–*R* glass relative to its Na*K*–*R* counterpart (Table 2). This is attributed primarily to the sharing of many NBO sites in the MgNa*K*–*R* structure between Na^+^ and Mg^2+^, coupled with the tighter control of Mg^2+^ to maintain a short Mg–O^[1]^ bond (≈203 pm; Table 2), which lengthens (Na–O^[1]^) by 5–8 pm relative to the bond length of ≈240 pm of the Mg-free glasses.

### 3.3 MD-Derived {NaO_*p*_} and {MgO_*p*_} Speciations

**Figure 2:**
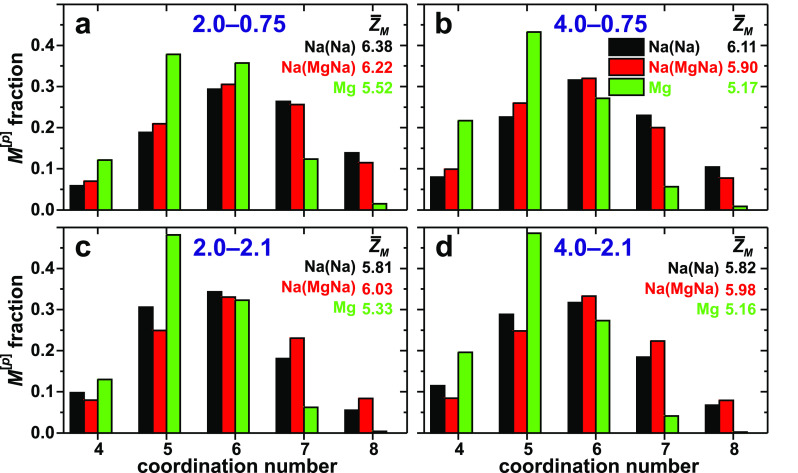
MD-derived distributions of Na^[*p*]^ (black and red bars) and Mg^[*p*]^ (green bars) coordination numbers in each Na*K*–*R* and MgNa*K*–*R* glass with (a) {*K*, *R*} = {2.0, 0.75}, (b) {*K*, *R*} = {4.0, 0.75}, (c) {*K*, *R*} = {2.0, 2.1}, and (d) {*K*, *R*} = {4.0, 2.1}. The black and red bars represent the coordination numbers of Na in the Na*K*–*R* and MgNa*K*–*R* (“MgNa”) glasses, respectively. The legends specify the average coordination numbers  and  of Na^+^ and Mg^2+^, respectively.

Figure 2 plots the distributions of {Na^[*p*]^} and {Mg^[*p*]^} coordinations observed from the glass models. As expected, the larger Na^+^ cation exhibits higher coordination numbers than Mg^2+^, which is mirrored in *average* coordination numbers  ranging over  and  and corresponding distributions peaking at Na^[6]^ and Mg^[5]^. Along previous findings from (boro)phosphosilicate glasses,^56,64,87^ substantial Na^[5]^ and Na^[7]^ populations are also present throughout all BS glass models (Figure 2). In contrast, the {Mg^[*p*]^} ensemble is more strongly peaked at *p* = 5, although significant contributions from MgO_4_ and MgO_6_ polyhedra are encountered in all structures, with the NaMg2.0–2.1 glass revealing nearly equal Mg^[5]^ and Mg^[6]^ populations. The herein modeled  values accord well with those observed by Pedone and co-workers from NBO-rich phosphosilicate^86^ and aluminoborosilicate^83^ glasses, all of which are higher than those reported in ref (85). Potential relationships between / and the glass compositions are discussed in section S1. The less dispersed distribution of {Mg^[*p*]^} populations relative to {Na^[*p*]^} stems from the larger CFS_Mg_ and the higher capacity of Mg^2+^ to control its coordination shell, as also mirrored in the respective standard deviation  of the *p*-distribution around its average value : it spans  for Na^+^ but is consistently smaller for Mg^2+^ () in the MgNa*K*–0.75 glasses and yet lower in their NBO-rich MgNa*K*–2.1 counterparts:  (Table 3).

**Table 3:** Distributions of Na^**[***p***]**^ and Mg^**[***p***]**^ Coordinations^a^

			100*x*_Na_^[*p*]^ (%)								100*x*_Mg_^[*p*]^ (%)					
glass	Z̅_Na_	*σ*_Na_^[*p*]^	4	5	6	7	8	9	Z̅_Mg_	*σ*_Mg_^[*p*]^	3	4	5	6	7	8
***R* = 0.75**																
Na2.0–0.75	6.38	1.30	5.9	18.8	29.4	26.4	13.9	4.1								
MgNa2.0–0.75	6.22	1.27	7.0	21.0	30.5	25.6	11.4	3.1	5.52	0.93	0.5	12.1	37.8	35.7	12.4	1.5
Na4.0–0.75	6.11	1.27	8.0	22.6	31.6	23.0	10.5	2.6								
MgNa4.0–0.75	5.90	1.26	9.9	26.0	32.0	20.0	7.8	1.8	5.17	0.91	1.4	21.7	43.2	27.2	5.6	0.9

***R* = 2.1**																
Na2.0–2.1	5.81	1.11	9.8	30.6	34.3	18.1	5.5	1.0								
MgNa2.0–2.1	6.03	1.18	8.0	24.9	33.0	23.0	8.4	1.8	5.33	0.79	0.0	13.0	48.2	32.3	6.2	0.4
Na4.0–2.1	5.82	1.20	11.5	28.8	31.7	18.4	6.8	1.5								
MgNa4.0–2.1	5.98	1.20	8.5	24.8	33.3	22.3	7.9	1.7	5.16	0.80	0.2	19.6	48.6	27.3	4.1	0.2
σ^b^	0.02	0.01	0.3	0.4	0.4	0.5	0.2	0.2	0.02	0.02	0.2	1.1	1.1	0.9	0.7	0.2

a MD-derived average coordination number of Na^+^ and Mg^2+^ () across the respective {Na^[*p*]^} and {Mg^[*p*]^} distributions, where  and  denote the corresponding fractional populations of the Na^[*p*]^ and Mg^[*p*]^ coordination species and  is the distribution width.

b The data uncertainties are ±1σ, with σ given for each entity.

The herein observed  values from a rather modest Na–(Mg)–B–Si–O glass ensemble agree well with those of previous MD-derived results gathered from large sets of Na-bearing borate, boro(phospho)silicate, and phosphosilicate glasses.^56,64^ Notably, the Ca^2+^ cation with an intermediate CFS between Na^+^ and Mg^2+^ (Table S1) reveals  values close to and only marginally lower than those of Na^+^. However, the  range is closer to that of Mg^2+^, spanning 0.8–0.9 in Na–Ca–Si–P–O glasses^64^ and 0.8–1.1 in their B-bearing counterparts.^56^ A general tendency is that both  and  values increase concomitantly with the B content of the glass (see Table 3 and ref (56)), whereas our rather sparse data available for Mg^2+^ merely suggests that  remains nearly constant for fixed-*K* MgNa*K*–*R* glasses but depends on *R*, i.e., on the NBO content.

In oxide-based glasses,  decreases for increasing *M*^*z*+^ CFS values. For instance, all high-CFS La^3+^, Y^3+^, Lu^3+^, and Sc^3+^ cations (Table S1) manifest narrow *p*-distributions in AS glasses.^5,88,89^ Indeed, although the La^3+^ ion is markedly larger than Mg^2+^—as is reflected in MD-derived average coordination numbers of 6.0–6.6 in La_2_O_3_–Al_2_O_3_–SiO_2_ glasses^88^— CFS_La_ = 0.52 Å^–2^ is slightly higher than CFS_Mg_ = 0.46 Å^–2^, yielding a coordination spread of . Incidentally, the Sc^3+^ cation with a substantial CFS of 0.68 Å^–2^ reveals very similar {Sc^[*p*]^} populations in Sc_2_O_3_–Al_2_O_3_–SiO_2_ glasses compared to the {Mg^[*p*]^} populations of the present MgNa*K*–*R* glasses (Figure 2), with  and  spanning 5.1–5.4 and 0.68–0.71, respectively (see Figure 3 of Okhotnikov et al.^89^).

A frequently suggested but hitherto unsettled issue concerns the possibility that a subensemble of Mg^2+^ does *not* act as a network *modifier* but merely assumes a network-*forming* role in the guise of MgO_4_ tetrahedra.^22,90−92^ Yet, discriminating between those two distinct structural scenarios is not straightforward even from glass models because precise and reliable criteria are difficult to formulate given that *all* electropositive *M*^*z*+^ cations coordinate a significant number of BO sites at the SiO_4_ and BO_*p*_ moieties (section 3.4). Table 3 reveals that Mg^[4]^ accounts for 12–22% of the Mg speciations of the present BS glasses. Even *if* {Mg^[4]^} would assume a *partial* network-forming role, it is likely to be only minor. Indeed, eq 9 rests on the *assumption* that *all* Na^+^/Mg^2+^ cations act as modifiers, where the excellent agreement between the MD-derived NBO populations and those obtained experimentally via eq 9 (Table 1) suggests that a vast majority of the Mg^2+^ cations (if not all) assume the expected network-modifying role. To widen the perspective, non-negligible Na^[4]^ populations are *also* predicted in MD-derived oxide-glass models (see Table 3 and refs (56) and (87)), but a potential network-forming capacity of the archetypal Na^+^*modifier* has to our knowledge not yet been suggested.

### 3.4 CFS-Dependent Preferences for *M*–BO/NBO Bonding

We now examine the MD-derived BO/NBO partitioning in the first coordination shells of the Na^+^ and Mg^2+^ cations in each BS glass structure, i.e., the distribution of the O^[2]^/O^[1]^ coordinations of the respective {NaO_*p*_} and {MgO_*p*_} ensembles presented in Table 4. Because CFS_Ca_ is intermediate between CFS_Na_ and CFS_Mg_ (Table S1) and the relative preferences for *M*^[*p*]^–BO/NBO bonding are strongly CFS-dependent, Table 4 also includes modeled data from *R*[0.5CaO–0.5Na_2_O]–B_2_O_3_–*K*SiO_2_ glasses, denoted “CaNa*K*–*R*” and discussed in refs (22), (50), and (51). Each NBO and BO fraction in the first coordination shell of an *M*^*z*+^ cation is denoted by *x*(*M*–NBO) and *x*(*M*–BO), respectively. It was determined from the MD-derived glass model, whereupon the corresponding *preferences* for *M*–NBO and *M*–BO bonding were calculated by *P*(*M*–NBO) = *x*(*M*–NBO)/*x*_NBO_, and *P*(*M*–BO) = *x*(*M*–BO)/*x*_BO_, with *x*(*M*–NBO) + *x*(*M*–BO) = 1.^56,93^ For *nonpreferential M*–BO/NBO bond formation, *P*(*M*–NBO) = *P*(*M*–BO) = 1 and the fractions of *M*–NBO and *M*–BO contacts match the respective *x*_NBO_ and *x*_BO_ values of the glass. The cases *P*(*M*–O^[*q*]^) > 1 and *P*(*M*–O^[*q*]^) < 1 mark the *preference* and *reluctance* of *M*–O^[*q*]^ formation, respectively, with the deviation from unity of *P*(*M*–O^[*q*]^) conveying the degree of preference/reluctance.

**Table 4:** MD-Derived Fractional Populations and Preferences for {Na, Ca, Mg}–{BO, NBO} Bonding^a^

		*x*(*M*–O^[1]^)			*P*(*M*–O^[1]^)			*x*(*M*–O^[2]^)			*P*(*M*–O^[2]^)		
glass	*x*_NBO_	Na	Ca	Mg	Na	Ca	Mg	Na	Ca	Mg	Na	Ca	Mg
***R* = 0.75**													
Na2.0–0.75	0.054	0.128			2.39			0.872			0.92		
CaNa2.0–0.75	0.083	0.114	0.408		1.37	4.91		0.886	0.592		0.97	0.65	
MgNa2.0–0.75	0.104	0.155		0.565	1.50		5.44	0.845		0.435	0.94		0.49

Na4.0–0.75	0.039	0.124			3.20			0.876			0.91		
CaNa4.0–0.75	0.064	0.131	0.446		2.05	6.95		0.869	0.554		0.93	0.59	
MgNa4.0–0.75	0.077	0.165		0.623	2.14		8.07	0.835		0.377	0.90		0.41

***R* = 2.1**													
Na2.0–2.1	0.364	0.537			1.48			0.463			0.73		
CaNa2.0–2.1	0.363	0.441	0.717		1.22	1.98		0.559	0.283		0.88	0.44	
MgNa2.0–2.1	0.382	0.477		0.808	1.25		2.11	0.523		0.192	0.85		0.31

Na4.0–2.1	0.228	0.419			1.84			0.581			0.75		
CaNa4.0–2.1	0.238	0.339	0.659		1.42	2.76		0.661	0.341		0.87	0.45	
MgNa4.0–2.1	0.253	0.368		0.784	1.45		3.10	0.632		0.216	0.85		0.29
σ^b^	0.001	0.003	0.010	0.007	0.02	0.09	0.07	0.003	0.010	0.007	0.01	0.01	0.01

a Fractional *x*(*M*–O^[1]^) and *x*(*M*–O^[2]^) populations out of the entire O speciation for *M* = {Na, Ca, Mg}, along with the corresponding preferences for *M*–O^[*p*]^ bonding defined by *P*(*M*–O^[*p*]^)=*x*(*M*–O^[*p*]^)/, where  and . The data from the CaNa*K*–*R* glasses were obtained from MD-generated models presented in ref.^50^

b The data uncertainties are ±1σ with σ given for each entity.

The data of Table 4 confirm the well-documented propensities of Na^+^ and Ca^2+^ cations to coordinate NBO rather than BO species^56,60,64,83,87,94−97^ and that *P*(*M*–NBO) increases concurrently with the *M*^*z*+^ CFS as follows:^56,64,86,87^*P*(Na–NBO) < *P*(Ca–NBO) < *P*(Mg–NBO). The precise *P*(*M*–NBO) value depends not only on the *M*^*z*+^*identity* but also on the {*R*, *K*} parameters of the BS glass: all Na^+^, Ca^2+^, and Mg^2+^ cations strongly prefer NBO coordination, which accentuates for (i) increasing *n*_Si_/*n*_B_ ratio (i.e., increasing *K*) and, in particular, (ii) decreasing *x*_NBO_ (i.e., decreasing *R*). Consequently, all three network-modifier species manifest the overall strongest preference for *M*–NBO bond formation in the {*K*, *R*} = {4.0, 0.75} structures (Table 4).

Trend (ii) is unsurprising, i.e., that the strongest preference for *M*–NBO bonding occurs in NBO-*poor* BS glasses. It conforms to the frequently encountered feature of oxide glasses in which their *propensity* for forming a given structural moiety deviates the most from that predicted by an unrestricted random/statistical distribution whenever its *abundance* is *low*. Some examples reported by us and others encompass the following: (I) the clustering of rare-earth cations in RE_2_O_3_–Al_2_O_3_–SiO_2_ (refs (93) and (98)) and Na_2_O–SiO_2_ (refs (99) and (100)) glasses (whereas if RE_2_O_3_ oxides are added to molten SiO_2_, strong RE^3+^ clustering occurs at *any* concentration^100,101^); (II) the deviations from an otherwise essentially random spatial distribution of  anions in Ca–Na–P–Si–O glasses occur for low P contents (*x*_P_ ≲ 0.015), which manifest a *minor* P–P aggregation.^102,103^ (III) The *preference* for each of the two prevalent P–O–**B**^[4]^ and P–O–**Si** linkages in borophosphosilicate glasses is *strongest* in Si-rich and B-rich glass structures, respectively, i.e., when the accompanying fractions of P–O–**Si** and P–O–**B**^[4]^ linkages dominate.^55^ Exceptions to this crude “rule of thumb” do indeed exist, such as that the spatial distribution of Na^+^ cations in Na_2_O–(CaO)–B_2_O_3_–SiO_2_ glasses, which is most uniform in Na-poor compositions but randomizes in modifier-richer glasses.^57^

The stronger preference for NBO coordination of the higher-CFS Ca^2+^ and Mg^2+^ ions compared to that of Na^+^ (refs (56), (64), (83), (86), (87), and (96)) is also mirrored in the reduced *P*(Na–NBO) value in each MgNa*K*–*R* glass relative to that its ternary Na*K*–*R* analog, notwithstanding that some of the mixed-cation glasses manifest slightly larger *x*(Na–NBO) *fractions* than their Na*K*–*R* counterparts due to their higher NBO contents accompanying Mg^2+^ introduction (Table 4). Yet, except for the overall NBO-richest {*K*, *R*}={2.0, 2.1} glasses (*x*_NBO_ ≈ 0.36), the low-CFS Na^+^ ion consistently features more BO than NBO contacts throughout all *R* = 0.75 glasses, for which NBO species accounts only for 11–17% of all Na–O bonds in each {NaO_*p*_} ensemble. That contrasts sharply with the {MgO_*p*_} speciation of the MgNa4.0–0.75 glass, for which NBO anions constitute 62% of all Mg–O^[*q*]^ bonds *despite* the low NBO abundance (*x*_NBO_ = 0.077), which is readily rationalized by the 2–3 times stronger Mg–NBO than Na–NBO affinity (Table 4).

The *P*(*M*–BO) < 1 values observed in Table 4 throughout all BS glass models and all three network modifier species mirror the as-expected reluctance of *M*–BO bond formation, which is accentuated for increasing *M*^*z*+^ CFS values as follows: *P*(Na–BO) > *P*(Ca–BO) > *P*(Mg–BO). Although the much stronger propensity for *M*–NBO over *M*–BO contacts in oxide glasses is both intuitive and well documented by computational modeling,^56,60,64,83,86,87,94,95^ it is challenging to *quantify* the *x*(*M*–NBO) and *x*(*M*–BO) fractions using experiments. Yet, this was recently accomplished by exploiting double-resonance ^17^O{^23^Na} and ^17^O{^27^Al} NMR applied to an AS glass of composition 10.8Na_2_O–32.3CaO–13.0Al_2_O_3_–44.0SiO_2_.^96^ That yielded estimated *x*(*M*–BO)/*x*(*M*–NBO) ratios of 1.4 and 0.37 for Na^+^ and Ca^2+^, respectively,^96^ incidentally very close to those of 1.3 (Na^+^) and 0.40 (Ca^2+^) predicted herein for the *M*–BO/NBO partitioning of the NaCa2.0–2.1 glass (Table 4). However, given the *by definition* very *local* structural information encoded by the Na–O^[*q*]^ contacts of the NaO_*p*_ polyhedra—and *M*–O^[*q̅*]^ bonds in general—coupled with the *direct scaling* of the *x*(Na–NBO) and *x*(Na–BO) fractions with the NBO content of the glass (e.g., see Table 4 and ref (56)), we discourage attempts to draw even qualitative conclusions about medium-range glass-structure features from {*x*(*M*–O^[*p*]^)} data alone, encompassing inferences about the spatial Na^+^ distribution and its *possible* implications for the (sub)nanometer-scale glass organization.^96,97^

### 3.5 B^[*p*]^/Si Intermixing Probed by ^11^B{^29^Si} REDOR NMR and MD Simulations

#### 3.5.1 Relative Degrees of B^[3]^–O–Si and B^[4]^–O–Si Bonding

**Figure 3:**
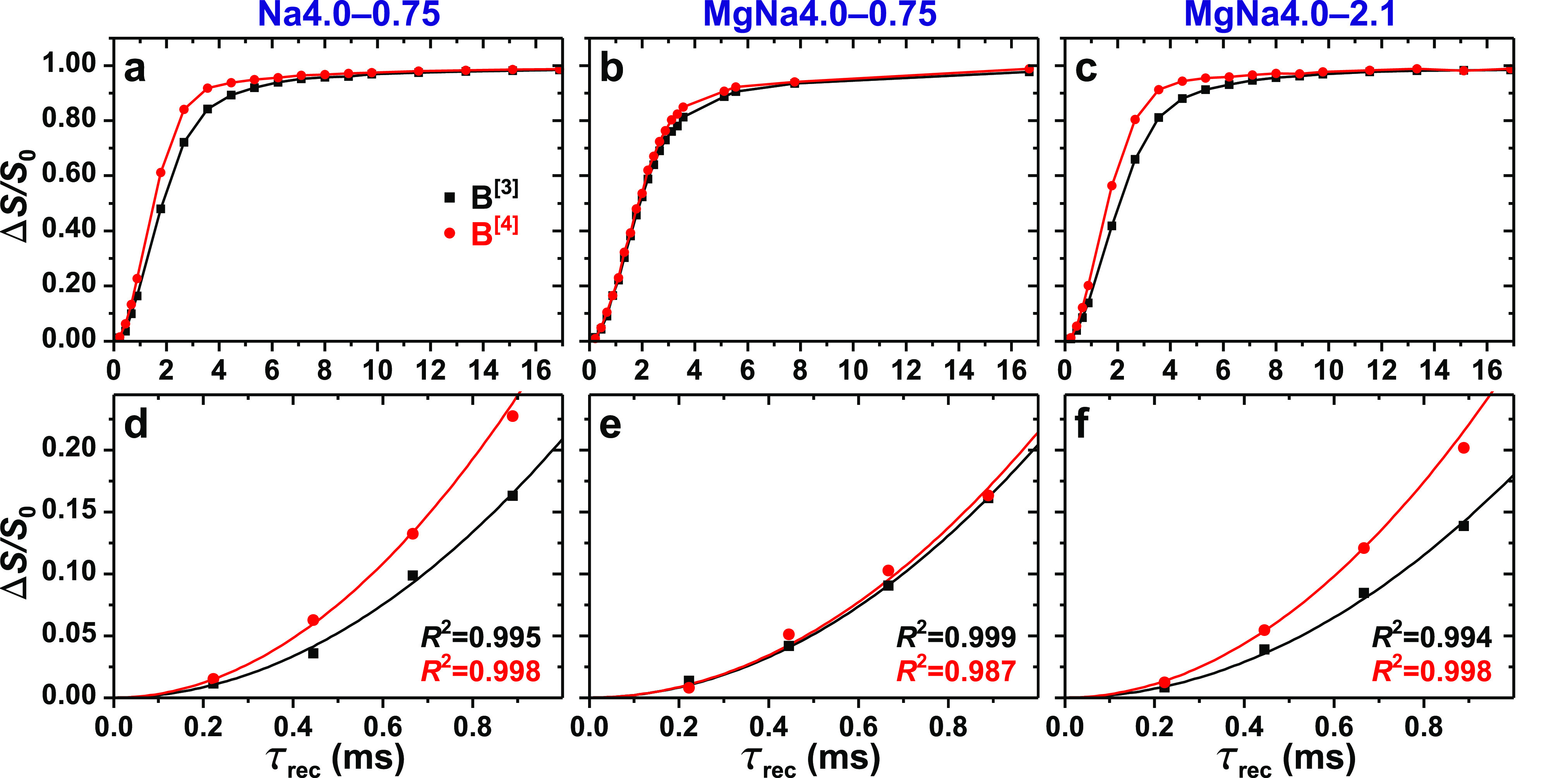
^11^B{^29^Si} REDOR NMR dephasing data (Δ*S*/*S*_0_) plotted against the recoupling/dephasing interval (τ_rec_) and recorded at 14.1 T and 9.00 kHz MAS from the (a, d) Na4.0–0.75, (b, e) MgNa4.0–0.75, and (c, f) MgNa4.0–2.1 glasses. The top panels (a–c) displays the entire ^11^B^[3]^ and ^11^B^[3]^ dephasing curves, whereas the bottom panels (d–f) show zoomed-in views of the initial dephasing regimes. Note that the lines in (a–c) only serve to guide the eye, while those of (d–f) are best-fit results to eq 7 for the data Δ*S*/*S*_0_ ⩽ 0.20. All data uncertainties are within the symbol sizes.

The relative extents of B^[3]^–O–Si and B^[4]^–O–Si bonding in the Na4.0–0.75, MgNa4.0–0.75, and MgNa4.0–2.1 structures were assessed by ^11^B{^29^Si} REDOR NMR experiments. Figure 3 displays the “dephasing” responses observed from the ^11^BO_3_ and ^11^BO_4_ resonances for increasing “dipolar recoupling” periods (τ_rec_) of ^11^B^[*p*]^–^29^Si through-space interactions that are responsible for the NMR-signal dephasing.^65,104^ While the ^11^B^[*p*]^-resonance dephasing is unaffected by any B^[*p*]^–O–B^[*q*]^ or Si–O–Si linkage of the structure, its rate increases concurrently with the number of ^11^B^[*p*]^–O–^29^Si linkages, thereby accelerating the progress toward the Δ*S*/*S*_0_ = 1 limit of “complete dephasing”^24,25,41,42^ (Figure 3a–c). As expected from the *higher*-coordination B^[4]^ sites and previous ^11^B{^29^Si} REDOR NMR reports from other BS glasses,^44,46^ their resonances reach the complete limit of complete dephasing well before their ^11^B^[3]^ counterparts (Figure 3a–c), except for the MgNa4.0–0.75 glass (vide infra).

Numerical fitting of the initial ^11^B^[*p*]^ NMR-signal dephasing regime with “short” τ_rec_ values (Figure 3d–f) to eq 7 yields an estimate of the dipolar second moment *M*_2_(B^[*p*]^–Si), which reflects the “aggregate” ^11^B^[*p*]^–^29^Si contact in the structure^24,41,42^ and grows concomitantly with the net *number* of direct B^[*p*]^–O–Si bridges, *N*(B^[*p*]^–O–Si), i.e., the average number of Si atoms in the second coordination shell of the {B^[*p*]^} sites: *M*_2_(B^[*p*]^–Si) ≈ *N*(B^[*p*]^–O–Si). As highlighted by previous reports utilizing *M*_2_/dipolar-interaction-based NMR analyses to make inferences about bonding statistics/preferences,^34,42,64^ however, this relationship is only approximate because *M*_2_(B^[*p*]^–Si) involves a sum over *all r*(B^[*p*]^–O–Si) distances in the structure (eq 6). Although the analysis of section S2 reveals that *M*_2_(B^[*p*]^–Si) approximates well the targeted information about the (average) number of direct B^[*p*]^–O–Si linkages, other factors degrade quantitative assessments of B^[4]^–O–Si bonding relative to B^[3]^–O–Si, which becomes underestimated by ≈25%.

Table 5 compiles the experimental *M*_2_(B^[*p*]^–Si) data together with those extracted from the glass models via eq 6. The consistently lower experimental *M*_2_(B^[*p*]^–Si) values compared to their MD-derived counterparts (by 40–55%) stem primarily from experimental imperfections, in particular rf inhomogeneity.^66,67^ Consequently, we focus our comparisons on the *relative M*_2_(B^[*p*]^–Si) trends among the B^[3]^/B^[4]^ coordinations encoded by each NMR- and MD-derived dipolar second-moment *ratio* (section S2):
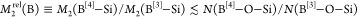
10Table 5 reveals a very good agreement between the experimental and modeled (B) values, whose deviations only amount to a few percent. Even the largest discrepancy observed for the MgNa4.0–0.75 structure (16%) must be considered decent in view of the large number of potential error sources that could degrade the agreement, notably those of the MD-generated glass *models*.

**Table 5:** ^11^B^**[***p*]^–^29^Si Dipolar Second Moments and *F*–O–*F*′ Bonding Preferences^a^

	*M*_2_(B^[3]^–Si)	*M*_2_(B^[4]^–Si)		*M*_2_(Si–B^[3]^)	*M*_2_(Si–B^[4]^)^b^		*P*(Si–O–*F*)^c^			*P*(B^[3]^ −O–*F*)^c^		*P*(B^[4]^–O–*F*)^c^
glass	(10^4^ Hz^2^)	(10^4^ Hz^2^)	*M*_2_^rel^(B)	(10^4^ Hz^2^)	(10^4^ Hz^2^)	*M*_2_^rel^(Si)	Si	B^[3]^	B^[4]^	B^[3]^	B^[4]^	B^[4]^
***R* = 0.75**												
Na2.0–0.75	5.13	7.58	1.48	9.32	16.61	1.78	0.95	0.90	1.16	0.66	1.38	0.50
MgNa2.0–0.75	5.41	7.16	1.32	14.02	10.14	0.72	0.99	0.99	1.03	0.81	1.27	0.54
Na4.0–0.75	7.08	9.51	1.34	6.70	10.04	1.50	0.99	0.94	1.09	0.76	1.38	0.40
	3.92 ± 0.05	5.65 ± 0.05	1.44 ± 0.05	3.00 ± 0.04	6.99 ± 0.06	2.33 ± 0.08						
MgNa4.0–0.75	7.23	8.85	1.22	10.12	5.33	0.53	1.00	1.01	0.99	0.74	1.33	0.49
	3.84 ± 0.03	4.02 ± 0.11	1.05 ± 0.06	5.30 ± 0.04	2.50 ± 0.07	0.47 ± 0.03						

***R* = 2.1**												
Na2.0–2.1	3.47	6.72	1.94	7.68	12.04	1.57	0.92	0.99	1.19	0.79	1.15	0.52
MgNa2.0–2.1	3.78	6.51	1.72	9.63	9.49	0.99	0.97	0.99	1.11	0.81	1.20	0.54
Na4.0–2.1	5.14	8.84	1.72	4.04	10.75	2.66	0.96	0.95	1.14	0.70	1.28	0.46
MgNa4.0–2.1	5.55	8.45	1.53	6.18	7.51	1.22	0.99	0.98	1.07	0.72	1.29	0.49
	3.37 ± 0.04	5.11 ± 0.04	1.51 ± 0.04	3.75 ± 0.04	4.55 ± 0.04	1.21 ± 0.03						
σ^d^	0.04	0.05	0.02	0.10	0.12	0.04	0.01	0.01	0.01	0.05	0.02	0.03

a Heteronuclear ^11^B^[*p*]^–^29^Si dipolar second moments, *M*_2_(B^[*p*]^–Si), calculated from the MD-derived glass model (eq 6) or obtained by fitting ^11^B{^29^Si} REDOR NMR data to eq 7 (data on line below the glass entry). (B) = *M*_2_(B^[4]^–Si)/*M*_2_(B^[3]^–Si).

b Calculated from eq 11 by using the {*M*_2_(B^[*p*]^–Si)} data. (Si) =*M*_2_(Si–B^[4]^)/*M*_2_(Si–B^[3]^).

c Preference factor *P*(*F*–O–*F*′) =*P*(*F*′–O–*F*) for *F*–O–*F*′ linkage formation of {*F*, *F*′} = {B^[3]^, B^[4]^, Si}. *P*(*F*–O–*F*′) is defined as the ratio between the as-observed number of *F*–O–*F*′ linkages in the glass model relative and that predicted from nonpreferential (statistical) {*F*, *F*′} intermixing.

d The uncertainties of the MD-derived data are ±1σ, with σ given for each entity.

For a statistical/nonpreferential *F*–O–*F*′ linkage-formation among {*F*, *F*′}={Si, B^[3]^, B^[4]^} in a BS structure *devoid* of NBO species, (B) is given by (stat) ≈ 1.18 (section S2). The MD derived (B) data, and notably the experimental counterparts, are markedly larger than (stat) for all glasses but MgNa4.0–0.75, in particular for the NBO-rich *R* = 2.1 members (Table 5). Two structural factors account for these observations:

(i) The NBO partitioning among Si, B^[3]^, and B^[4]^ species in BS glasses was discussed previously,^34,50,56^ suggesting a substantially stronger propensity for B^[3]^–NBO bonding relative to B^[4]^–NBO. Except for *very* NBO-rich glasses,^105^ the latter is even considered “forbidden” by most scientists in the field^27,69,106−108^ but remains frequently observed to minor extents in numerous MD-derived glass models.^34,55,56,75,77,82^ Consequently, a progressively growing NBO accommodation at the BO_3_ groups for increasing *x*_NBO_ reduces the possibility of *any* B^[3]^–O–*F* linkage type, thereby boosting the (B) values of all NBO-rich glasses (Table 5), while the *relative* dipolar second moment (eq 10) is independent of the degree of Si–NBO bonding.
(ii) The second effect underlying the increased NMR/MD-derived (B) values in *any* BS glass is less influential but stems from a slightly higher preference for B^[4]^–O–Si bridges than B^[3]^–O–Si linkages. Owing to the difficulties in quantifying the *preference factor P*(*F*–O–*F*′) for *F*–O–*F*′ bond formation by experiments, however, current quantitative insights stem dominantly from computational modeling;^55−57,64,109^ see ref (50) for experimental attempts to estimate the {*P*(B^[*p*]^–O–B^[*q*]^)} subset. Table 5 lists the MD-derived {*P*(*F*–O–*F*′)} factors of the present BS glasses. Here, *P*(*F*–O–*F*′) = 1 denotes a strictly nonpreferential *F*/*F*′ intermixing, whereas *P*(*F*–O–*F*′) > 1 and *P*(*F*–O–*F*′) < 1 imply a preference and reluctance for *F*–O–*F*′ linkage formation, respectively. Although the bonding preferences depend slightly on the glass composition, the trends of Table 5 conform well to those discussed previously for Na/Ca-bearing boro(phospho)silicate glasses,^34,50,55,56^ revealing the strongest preference (reluctance) for BO_3_–BO_4_ (BO_4_–BO_4_) pairs. While B^[3]^–O–B^[3]^ linkages are also disfavored, *all* remaining Si–O–{Si, B^[3]^, B^[4]^} linkages form nearly statistically, i.e., each fractional population is given roughly by the product of the respective {*x*_Si_, , } molar fractions in the glass structure. Nonetheless, along previous findings from boro(phospho)silicate glasses,^34,50,55,56^Table 5 conveys the following subtle trend: *P*(Si–O–B^[4]^) > *P*(Si–O–B^[3]^) ≈ *P*(Si–O–Si) ≈ 1 [note that *P*(*F*–O–*F*′) = *P*(*F*′–O–*F*)]. Hence, the slightly stronger propensity for forming B^[4]^–O–Si linkages relative to B^[3]^–O–Si underlies the observed (B)(stat) trend, notably so for the ternary Na*K*–*R* glasses (sections 3.6 and S2).

#### 3.5.2 B^[4]^ Environments with Variable Numbers of Si and B Neighbors

**Figure 4:**
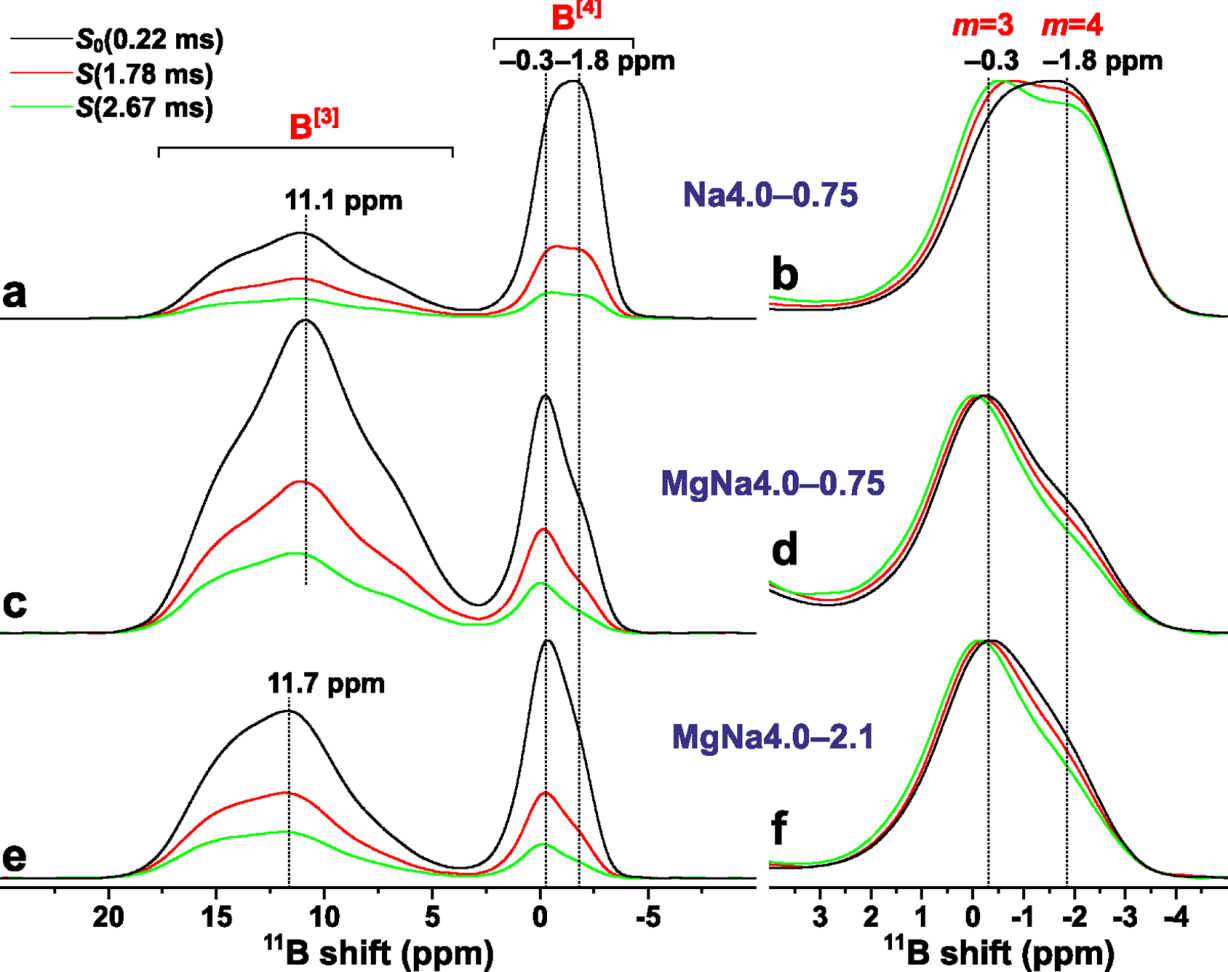
Selected ^11^B{^29^Si} REDOR NMR spectra of the (a, b) Na4.0–0.75, (c, d) MgNa4.0–0.75, and (e, f) MgNa4.0–2.1 glasses for the as-indicated dipolar recoupling periods (τ_rec_). (a, c, e) REDOR reference spectra [*S*_0_(0.22 ms)] shown together with the REDOR NMR spectra [*S*(τ_rec_)] obtained for long dephasing intervals. (b, d, f) Zoomed-in view of the ^11^B^[4]^ NMR spectral region (normalized to unity maximum intensity throughout), where two peaks attributed to ^11^ B^[4]^(4Si) and ^11^B^[4]^(3Si) moieties are marked as *m* = 4 and *m* = 3, respectively.

Figure 4a, c, and e shows the REDOR “reference” ^11^B NMR spectrum, *S*_0_(τ_rec_), of each Na4.0–0.75, MgNa4.0–0.75, and MgNa4.0–2.1 glass recorded at the shortest τ_rec_ = 0.22 ms value, along with two REDOR spectra [*S*(τ_rec_)] observed for long dephasing periods of τ_rec_={1.78, 2.67} ms. As expected from the Δ*S*/*S*_0_ dephasing data of Figure 3, the latter spectra manifest progressively diminished ^11^B^[3]^ and (particularly) ^11^B^[4]^ resonance intensities for increasing τ_rec_, except for the MgNa4.0–0.75 specimen, which reveals comparable NMR-signal decays.

We now focus on the ^11^B^[4]^ NMR-signal dephasing, which is more pronounced for the low-δ_B_ spectral region <−1 ppm, as is most transparent from the normalized NMR spectra presented in Figure 4b, d, and f. Along the ^11^B NMR spectral deconvolution results of the Na4.0–0.75 and NaMg4.0–0.75 glasses presented by Lv et al.,^51^ the two peak components at ≈ −1.8 ppm and ≈ −0.3 ppm are attributed to B^[4]^(OSi)_4_ and B^[4]^(OSi)_3_(OB) moieties, respectively,^35−37,40,72^ which are abbreviated as B^[4]^(4Si) and B^[4]^(3Si). Owing to its larger number of Si neighbors, the resonance decay of ^11^B^[4]^(4Si) is stronger than that of its ^11^ B^[4]^(3Si) counterpart. To adequately deconvolute the ^11^B^[4]^ NMR signal region of the two Na4.0–0.75 and MgNa4.0–0.75 glasses, however, it was necessary to also include a minor peak at ≈1.2 ppm from ^11^B^[4]^(2Si) environments (accounting for 9% and 17% out of all B^[4]^(*m*Si) groups, respectively).^51^ These NMR signals are not clearly discernible in the REDOR spectra, but they are expected to decay even slower than the ^11^B^[4]^(3Si) resonance, as is also hinted in Figure 4.

### 3.6 Effects from Mg^2+^ on the B^[*p*]^/Si Intermixing

Although all herein discussed BS glasses but MgNa4.0–0.75 manifest a markedly larger number of B^[4]^–O–Si linkages than B^[3]^–O–Si bridges, the partial replacement of Na^+^ by Mg^2+^ leads to a non-negligible reduction of (B) throughout: contrasting the MD-derived *M*_2_(B^[4]^–Si) and *M*_2_(B^[3]^–Si) values of the Na*K*–*R* glass and its MgNa*K*–*R* counterpart in Table 5 reveals that the decrease of (B) stems from an increase of *M*_2_(B^[3]^–Si) at the expense of *M*_2_(B^[4]^–Si), altogether implying that Mg^2+^-for-Na^+^ substitution is accompanied by an increase (decrease) in the number of B^[3]^–O–Si (B^[4]^–O–Si) linkages. The predictions of the Na4.0–0.75 and MgNa4.0–0.75 glass models are corroborated by the REDOR NMR experiments, which reveal a significant reduction in *M*_2_(B^[4]^–Si) for the MgNa4.0–0.75 glass ((B) = 1.05) relative to (B) = 1.44 for Na4.0–0.75 (yet the experimental *M*_2_(B^[3]^–Si) values of both glasses are nearly equal; Table 5). These effects are evident from the nearly coincident ^11^B^[*p*]^{Si} REDOR NMR dephasing curve observed for MgNa4.0–0.75 (Figure 3b,e), in sharp contrast to those of the other two glasses for which the ^11^B^[4]^ NMR-signal dephasing rate consistently exceeds that of ^11^B^[3]^.

We stress that although a lower total number of B^[4]^–O–Si linkages upon Mg^2+^ incorporation is indeed anticipated from the drastic decrease of the BO_4_ population alone (Table 5), that effect is inconsequential for *M*_2_(B^[4]^–Si) because its value is *independent* on {, } (eq 6), in contrast to *M*_2_(Si–B^[4]^); see section 3.7. Notably, the MD-generated preference factors of Table 5 rationalize these *quantitative* trends of dipolar second moments and the number of B^[*p*]^–O–Si bonds as *originating* from the more fundamental feature of a *decrease* in *P*(B^[4]^–O–Si) upon the introduction of the high-CFS Mg^2+^ cation, which for all NBO-poor (Mg)Na*K*–0.75 glasses is moreover emphasized by a concomitant *increase* of *P*(B^[3]^–O–Si) (see section S2).

**Figure 5:**
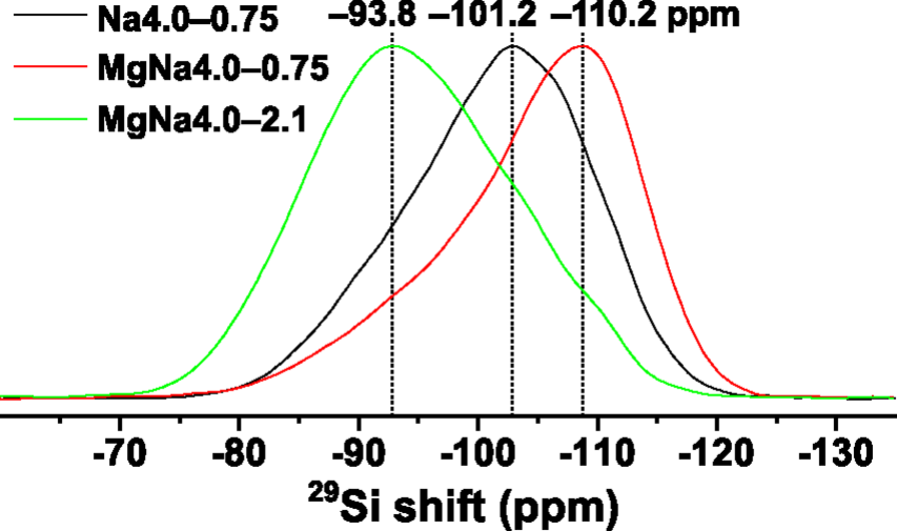
^29^Si MAS NMR spectra recorded at 9.4 T and 14.00 kHz MAS from the Na4.0–0.75, MgNa4.0–0.75, and MgNa4.0–2.1 glasses. The higher structural complexity of the NBO-rich MgNa4.0–2.1 structure is mirrored in its ≈5 ppm wider resonance relative to that of its MgNa4.0–0.75 counterpart with a low NBO content, along with a more deshielded chemical shift at the peak maximum (i.e., a higher value of δ_Si_).

The weakened B^[4]^/Si contacts upon Mg^2+^-for-Na^+^ substitution reflect a general trend of a linearly decreasing fraction of B^[4]^–O–Si linkages (out of all B^[4]^–O–Si/B bridges) for increasing CFS_*M*_, as deduced from ^11^B^[4]^ MAS NMR spectra deconvolutions.^51^ These CFS effects are also mirrored in the ^29^Si MAS NMR spectra of the Na4.0–0.75, MgNa4.0–0.75, and MgNa4.0–2.1 glasses shown in Figure 5: as expected from amorphous (boro)silicates, the net ^29^Si resonance is broad but consistent with solely ^29^SiO_4_ groups.^24,82,110−113^ Hence, the ^29^Si MAS NMR information content is very limited, *at best* offering *qualitative* inferences.^113,114^ Even for the (almost) NBO-free Na4.0–0.75 and MgNa4.0–0.75 glasses, the net ^29^Si NMR peak stems from a plethora of unresolved ^29^**Si**(OB^[3]^)_*p*_(OB^[4]^)_*q*_(OSi)_4–*p*−*q*_ resonances.

The ^29^Si chemical-shift dispersion and the precise value of the *most probable* shift  located at the maximum NMR-peak intensity depend predominantly on the {Si, B^[3]^, B^[4]^} distribution in the second coordination sphere of ^29^Si, where a 3–5 ppm increase of δ_Si_ accompanies each ^29^**Si**–O–Si → ^29^**Si**–O–B^[4]^ bond replacement, whereas ^29^**Si**–O–Si → ^29^**Si**–O–B^[3]^ substitutions leave the ^29^Si chemical shift essentially invariant.^82,110−113^ Hence, the markedly reduced number of Si–O–B^[4]^ bonds in the MgNa4.0–0.75 structure and the concurrently increased number of Si–O–B^[3]^ and Si–O–Si linkages rationalize its net ^29^Si resonance-displacement toward lower shifts relative to Na4.0–0.75, while the evident high-ppm “tail” of the NMR stems from the few(er) remaining ^29^**Si**–O–B^[4]^ sites (Figure 5). The structural complexity is accentuated further in the NBO-rich(er) MgNa4.0–2.1 glass, which *additionally* exhibits variable numbers of Si–NBO bonds among the SiO_4_ groups. Besides rationalizing the broader ^29^Si NMR peak, it accounts for the 5.8 ppm higher  value relative to that observed for MgNa4.0–0.75 (Figure 5), stemming from a typically 7–12 ppm ^29^Si chemical-shift increase for each BO → NBO bond replacement.^82,110−113^

It is known that Si-rich but *M*^*z*+^-poor *M*_*z*/2_O–B_2_O_3_–SiO_2_ glasses exhibit compositional regions of liquid immiscibility, which widen for high-CFS *M*^*z*+^ cations.^37,115−119^ A glass-in-glass separation occurs upon cooling, typically identified as (or often merely *assumed* to involve) one Si-dominated phase coexisting with a B-rich borate/BS counterpart^37,115−118^ and manifested by ^29^Si MAS NMR peak displacement toward more negative chemical shifts^117^ near ≈ –110 ppm observed for vitreous SiO_2_.^113^ Incidentally, that is also observed for the MgNa4.0–0.75 glass (Figure 5) but is readily attributed to the significantly *fewer* Si–O–B^[4]^ bonds in the MgNa4.0–0.75 structure relative to Na4.0–0.75 (note that *both*^29^**Si**–O–Si/B^[3]^ environments resonate at near-equal shifts^113^). Notably, previous heteronuclear ^11^B/^29^Si NMR studies on the bearings from thermal annealing of BS glasses, which may induce structural inhomogeneities and/or phase separation, also reveal that the number of Si–O–B^[3]^ bonds increases relative to Si–O–B^[4]^,^45−48^ thereby mirroring the herein observed ^29^Si shielding accompanying Mg^2+^ incorporation into a Na_2_O–B_2_O_3_–SiO_2_ glass. However, backscatter scanning electron microscopy (SEM) images (not shown) did not indicate phase separation. Yet nanometer-scale inhomogeneities cannot be excluded, as they would remain undetected both over the ≳1 μm and <0.5 nm length scales probed by our SEM and NMR experimentation, respectively.

Remarkably, despite numerous reports on phase-separated BS glasses, the precise chemical compositions of the two (assumed) coexisting phases remain surprisingly poorly defined. Interestingly, all of the few studies using techniques that *directly* inform on the Si/B^[*p*]^ intermixing (i.e., heteronuclear NMR) of annealed/phase-separated BS glasses do *not* point toward a categorical separation into Si-rich and B-rich phases, but merely to a significant reduction of the number of Si–O–B^[4]^ linkages, while the number of Si–O–B^[3]^ bonds remains invariant, or even increases,^45−48^ within one, or several, *borosilicate* phase(s). A recently reported elemental analysis of a phase-separated Na BS glass did reveal two such phases with distinct Si and B contents; however, both contents were substantial in each phase.^119^ This issue should be investigated further to better define *what* phases coexist in BS glasses attributed to exhibit nanometer-range structural inhomogeneities.

### 3.7 Relative Degrees of Si–O–B^[3]^/B^[4]^ Bonding

This section discusses the dipolar second moments {*M*_2_(Si–B^[*p*]^)} listed in Table 5. They are accessible from the {*M*_2_(B^[*p*]^–Si)} set by the general expression *M*_2_(S–I) for S–I spin-pairs,^63^ which for *S* = 1/2 (^29^Si) and *I* = 3/2 (^11^B) evaluates to
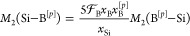
11where  for ^11^B, whereas ^29^Si constitutes ≈100% of all Si sites in the isotopically enriched glasses . Although the *M*_2_(Si–B^[*p*]^) and *M*_2_(B^[*p*]^–Si) values are directly related for a given glass specimen, they nonetheless convey *complementary* information: while *M*_2_(B^[*p*]^–Si) is proportional to the number of B^[*p*]^–O–Si linkages at the BO_*p*_ ensemble, *M*_2_(Si–B^[*p*]^) relates to the number of Si–O–B^[*p*]^ bridges at {SiO_4_}.

The crucial utility of eq 11 is its straightforward route to derive *both M*_2_(Si–B^[3]^) and *M*_2_(Si–B^[4]^) values, whose separate estimation is very difficult to accomplish accurately with current state-of-the-art heteronuclear NMR methodology. For instance, the *less* informative *M*_2_(Si–B) entity, i.e., the *aggregate* dipolar second moment across the entire {B^[3]^, B^[4]^} ensemble, may be obtained from a ^29^Si{^11^B} REAPDOR NMR experiment,^104^ with the caveat that the experimental protocol along with its subsequent data analysis yield less accurate dipolar second-moment estimates than the *M*_2_(B^[3]^–Si) and *M*_2_(B^[4]^–Si) outcomes of ^11^B{^29^Si} REDOR NMR, from which moreover *both M*_2_(Si–B^[3]^) and *M*_2_(Si–B^[4]^) results are readily extracted via eq 11.^57,63,64^ Hence, *sole*^11^B{^29^Si} REDOR NMR application to a BS glass specimen unveils all four independent *M*_2_(B^[3]^–Si), *M*_2_(B^[4]^–Si), *M*_2_(Si–B^[3]^), and *M*_2_(Si–B^[4]^) results.

From its definition (eq 6), it follows that *M*_2_(B^[*p*]^–Si) is *independent* on , while *M*_2_(Si–B^[*p*]^) scales linearly with the B^[*p*]^ population of the glass but is independent of the Si content. That feature rationalizes the excellent agreement observed between the NMR/MD-derived (Si) ≡ *M*_2_(Si–B^[4]^)/*M*_2_(Si–B^[3]^) data for the Mg-bearing glasses of Table 5 (whose ,  sets accord very well), whereas the significantly underestimated MD-derived BO_4_ population of Na4.0–0.75 (section 3.1) accounts for the lower modeled (Si) result. The direct *M*_2_(Si–B^[*p*]^) dependence on  renders the (Si) ratio a very sensitive probe of the reduced Si–O–B^[4]^ bonding in the glass structure upon Mg^2+^ incorporation, as mirrored in the markedly lower {(Si)} values relative to their {(B)} counterparts (Table 5) and underscoring the time/effort-saving benefits of having *both* {*M*_2_(B^[*p*]^–Si)} and {*M*_2_(Si–B^[*p*]^)} data available from *one sole* NMR experiment and eq 11 (see refs (57), (63), and (64) for further examples.

Both (B) and (Si) scale approximately as  (eq 6 and section S2), while (Si) is additionally proportional to  (eq 11). These parameter ratios are listed in Table S4, along with those of *P*^rel^(Si) ≡ *P*(Si–O–B^[4]^)/*P*(Si–O–B^[3]^). Disregarding the MgNa4.0–0.75 glass with much weaker Si/B^[4]^ contacts, *P*^rel^(Si) > 1 holds throughout. When taken together with *N*^rel^(stat) = 1.44 reflecting a nonpreferential/statistical Si–O–B^[3]^/B^[4]^ bond formation (section S2), the product of the , *P*^rel^(Si), and  components is consistently slightly larger than (Si) but yields overall good predictions for all BS glasses (Table S4), despite that the crude approximations made are only expected to capture the *R* = 0.75 glasses with low NBO contents (section S2). The good predictions also observed for the *experimental*(Si) data are particularly gratifying because they assumed the *MD-derived* and *P*^rel^(Si) parameters.

Notably, although illustrated in the context of the *M*_2_(Si–B^[*p*]^) and (Si) entities, the various glass composition/structure parameters discussed above are readily replaced by their analogs underpinning any *M*_2_(*F*–*F*′) entity reflecting the number of *F*–O–*F*′ linkages at *F*O_*p*_ polyhedra that may interlink with two (or several) *F*O_*p*_, *F*′O_*p*′_, and *F*″O_*p*″_ polyhedral types.

### 3.8 Inferred Si/B^[*p*]^ Intermixing Versus Current BS Glass-Structure Descriptions

The findings herein of an overall larger extent of B^[4]^–O–Si than B^[3]^–O–Si bonding—yet with both linkage-types being abundant throughout all glasses (Table 5)—suggest BS glass networks with substantial {Si, B^[3]^, B^[4]^} intermixing. When combined with previous inferences of the coexistence of *all three* B^[3]^–O–B^[3]^, B^[3]^–O–B^[4]^ and B^[4]^–O–B^[4]^, linkages,^34,50^ the results portrays a network with all six *F*–O–*F*′ linkage-types among {Si, B^[3]^, B^[4]^} encountered in significant populations, each scaling with the molar fractions of its constituents but with higher-than-statistical numbers of the most preferred B^[3]^–O–B^[4]^ and Si–O–B^[4]^ bonds, while B^[4]^–O–B^[4]^ linkages are present but disfavored (Table 5).

Bray and co-workers introduced a structural description,^52,53^ herein referred to as the Yun–Dell–Bray–Xiao (YDBX) model, which accurately reproduces the experimental {, } fractions across the entire glass-formation region of the Na_2_O–B_2_O_3_–SiO_2_ system and has found widespread recognition, particularly within the NMR/glass community. Notwithstanding its success in predicting the borate speciation, the YDBX model attempts—from routine ^11^B NMR experimental information *alone—*to furnish a very detailed (but oversimplified) structural description, involving a (very) confined set of larger BO_*p*_/SiO_4_ molecular aggregates (“superstructural units”^27,70,120^). Superstructural units are indeed known to build many crystalline borate/BS phases,^27^ and numerous Raman studies support their existence for glasses as well.^28,110,120,121^ While BS-based glass structures most likely do comprise *some* larger BO_*p*_/SiO_4_ molecular aggregates, it is difficult to reconcile the main body of experimental reports with any dominating role thereof (except for limiting cases, such as vitreous B_2_O_3_^27,122,123^).

Indeed, ^11^B, ^29^Si, and ^17^O (3Q)MAS NMR reports suggest markedly *more disordered* BS networks than the (for a glass) exceptionally high medium-range order postulated by the YDBX model, notably its Si/B^[3]^/B^[4]^-intermixing predictions.^36,37,39,40,47,108,110,111^ We guide the reader to the thoughtful but critical remarks made by Möncke et al.^47^ Notably, the YDBX model predicts that only Si–O–Si/B^[4]^ and B^[3]^–O–B^[3]^/B^[4]^ bonds occur in the present Na*K*–0.75 glasses.^52,53^ Hence, Si–O–B^[3]^ are absent, in sharp *qualitative disagreement* with the results for any glass of Table 5, encompassing direct experimental proof of significant Si–O–B^[3]^ bonding in the Na4.0–0.75 glass, which is accentuated in the Mg-bearing glass structures. Furthermore, earlier findings from similar NMR experimentation compared to that employed herein suggested that SiO_4_ interlinks extensively with *both* BO_3_/BO_4_ moieties in BS glasses.^44,46−49^ The distinctly different Si/B^[*p*]^ intermixing predicted by the YDBX model and experimental/modeling findings herein and in refs (36), (37), (39), (40), and (46−48) stems from the YDBX-postulated but grossly underestimated degree of Si–O–B^[3]^ bonding (i.e., *P*(Si–O–B^[3]^) ≈ 0), whereas in fact *P*(Si–O–B^[3]^) ≲ *P*(Si–O–B^[4]^), yielding *P*(Si–O–B^[4]^)/*P*(Si–O–B^[3]^) ≈ 1.2 (Table 5). Analogous contradictions with many experimental findings also plague the alternative branch of “random-network model” (RNM) descriptions^69,106,107,124^ originating from Zachariasen and Warren,^125,126^ which overestimate the structural disorder by largely ignoring *F*–O–*F*′ bonding preferences (see section 3.5 and comments in ref (50)).

To reconcile the orthogonal implications for the structural order from the *too categorical* RNM^69,106,107,124^ and “superstructural-unit”^27,70,120^ borate/BS glass descriptions, we recently highlighted a “hybrid” model thereof.^50^ While we are unaware of previous explicit outlines or discussions of a BS glass network being built from some superstructural units *along with* near-randomly intermixed BO_3_/BO_4_/SiO_4_ groups across a <1 nm scale, even the well established and noncontroversial structure of vitreous B_2_O_3_ conforms to such a hybrid structural picture. Here, superstructural B_3_O_6_ units (boroxol rings, which comprise ≈70% of all B^[3]^ sites^27,123^) coexist with ring-*inter*linking BO_3_ groups.^27,122,123^ The introduction of network modifiers along with another network former (Si) naturally increases the structural disorder. This is, for instance, manifested by the strongly altered Si intermixing with both B^[3]^ and B^[4]^ accompanying the replacement of a low-CFS Na^+^ cation by Mg^2+^ (section 3.5) and also mirrored in B^[3]^–O–B^[3]^/B^[4]^ and B^[4]^–O–B^[4]^ populations somewhat closer to a statistical {B^[*p*]^–O–B^[*q*]^} intermixing in Mg-bearing BS glasses.^50^ Hence, all experimental/modeled results herein and in ref (50) as well as previous work^36,37,39,40,47−49,110,111^ are consistent with a hybrid random/superstructural-unit BS glass-network description, which nonetheless remains to be concretized from a quantitative standpoint.

## 4 Conclusions

We investigated the structural alterations occurring across a subnanometer scale when Mg^2+^ replaces Na^+^ in four *R*Na_2_O–B_2_O_3_–*K*SiO_2_ base glass compositions with *K* = *n*(SiO_2_)/*n*(B_2_O_3_) = {2.0, 4.0} and *R*=[*n*(MgO) + *n*(Na_2_O)]/*n*(B_2_O_3_) = {0.75, 2.1}. Na^+^ and Mg^2+^ exhibit average coordination numbers that vary across  and  among the glasses, where Na^[6]^ and Mg^[5]^ coordinations are most abundant. A non-negligible fraction (12–22%) of Mg^[4]^ species is observed but without evidence supporting a network-forming role of the MgO_4_ groups. The higher CFS of Mg^2+^ is manifested in narrower distributions of coordination numbers {Mg^[*p*]^} compared with {Na^[*p*]^}, as well as a 2–3 times stronger preference for NBO coordination relative to Na^+^. Although all *M*^*z*+^ species prefer *M*–NBO bonding over *M*–BO, the preference increases concurrently with the CFS along the series *P*(Na–NBO) < *P*(Ca–NBO) < *P*(Mg–NBO), where the precise preference factors also depend on the BS glass composition and, in particular, on the NBO content. Hence, whereas Na^+^, Ca^2+^, and Mg^2+^ all exhibit the *strongest affinity* for *M*–NBO bonding in the *R* = 0.75 glasses with low NBO contents (*x*_NBO_ < 0.1), the *fractions* of *M*–BO bonds are significant for all glasses/cations, amounting to roughly 86%, 57%, and 40% out of all BO/NBO species in the first coordination shells of Na^+^, Ca^2+^, and Mg^2+^, respectively.

The partial replacement of Na^+^ by Mg^2+^ induces primarily the following structural alterations:

(i) Significant BO_3_ → BO_4_ and BO → NBO conversions occur. Whereas BO_4_ accounts for 50–60% of the borate speciations of the Na_2_O–B_2_O_3_–SiO_2_ glasses, they only amount to 30–45% in the MgO–Na_2_O–B_2_O_3_–SiO_2_ structures.
(ii) The average Na–NBO bond length is 5–8 pm longer in the MgNa*K*–*R* glasses relative to that of ≈240 pm in the absence of Mg^2+^. That effect likely stems from the sharing of many NBO sites between Na^+^ and Mg^2+^, where the stronger capacity of the high-CFS cation to maintain its short Mg–NBO distance (≈203 pm) perturbs the local Na coordination environment.
(iii) All *R*Na_2_O–B_2_O_3_–*K*SiO_2_ glass structures reveal a clear *preference* for B^[4]^–O–Si linkages over B^[3]^–O–Si. Upon Mg^2+^-for-Na^+^ substitution, however, the preference for B^[4]^–O–Si is *reduced* (i.e., *P*(B^[4]^–O–Si) is decreased), whereas the propensity for B^[3]^–O–Si bridges either remain unaffected (for NBO-rich glasses; *R* = 2.1) or even increases (for NBO-poor glasses; *R* = 0.75). This results in *P*(B^[4]^–O–Si) ≈ *P*(B^[3]^–O–Si) for both MgNa2.0–0.75 and MgNa4.0–0.75 glasses, whereas B^[4]^–O–Si linkage formation remains (slightly) preferred for the *R* = 2.1 analogs. We stress that these bonding preferences are *independent* on the borate speciation. However, effects (i) and (iii) together account for effect (iv) below, namely
(iv) a significant reduction in the number of B^[4]^–O–Si bonds in the quaternary MgNa*K*–*R* glasses. The former bridges dominate throughout all Na_2_O–B_2_O_3_–SiO_2_ glasses, notably so for the NBO-rich members, where the stronger propensity for B^[3]^–NBO over B^[4]^–NBO bonding leads to glass networks with 1.7–2 times more B^[4]^–O–Si linkages compared with B^[3]^–O–Si, as estimated from dipolar second moments obtained from either ^11^B{^29^Si} REDOR NMR experiments or MD simulations.

The diminished B^[4]^–O–Si bonding upon Mg^2+^ incorporation is most evident for the MgNa4.0–0.75 glass network, which exhibits similar numbers of B^[4]^–O–Si and B^[3]^–O–Si linkages, whereas the Na4.0–0.75 analog features ≈1.4 times more B^[4]^–O–Si bridges than B^[3]^–O–Si. The lower number of B^[4]^–O–Si linkages in the MgNa4.0–0.75 structure is also mirrored in its ^29^Si MAS NMR spectrum, whose broad resonance is centered around a chemical shift close to that of vitreous SiO_2_ but stemming from ^29^**Si**–O–Si *as well as*^29^**Si**–O–B^[3]^ environments, both of which resonate at very similar chemical shifts.^82,110−113^ We found no indications of phase separation of the MgNa4.0–0.75 glass specimen, which most likely constitutes a single amorphous *borosilicate* phase with emphasized B^[3]^/Si contacts as compared to its NBO-richer MgNa4.0–2.1 counterpart or any Na*K*–*R* glass structure. The findings herein of reduced B^[4]^/Si contacts in the Mg-bearing glasses echo those deduced from heteronuclear ^11^B/^29^Si NMR experimentation on heat-treated BS glasses^45−48^ that are often taken to imply separation into Si and B dominated phases from face-value interpretations of routine infrared, Raman, or ^29^Si NMR spectra.

More detailed information about the borate environments and the Si/B^[*p*]^ intermixing require reliable ^11^B MAS NMR spectral deconvolutions, which are far from straightforward concerning the ^11^B^[3]^ resonances^22,113^ but could give more quantitative information about the *M*_2_(B^[4]^–Si) dipolar second moments for the B^[4]^(2Si), B^[4]^(3Si), and B^[4]^(4Si) environments that coexist in the glass and together yielding each average *M*_2_(B^[4]^–Si) value. This topic, along with the prospects for realistic ^11^B NMR spectral deconvolutions, will be discussed in upcoming publications.

## Supporting Information Available

Discussion on the glass-composition dependence of *Z̅*_Na_ and the relationship between the dipolar second moment *M*_2_(*F*–*F*′) and the number of *F*–O–*F*′ linkages in the glass; tables with cation field strengths, MD simulation parameters, the dependence on *M*_2_ on structural parameters, and MD-derived *F*–O–*F*′ bond lengths (PDF)
