# Supplementary material for: Sub-Nanometer-Range Structural Effects From Mg2+ Incorporation in Na-Based Borosilicate Glasses Revealed by Heteronuclear NMR and MD Simulations
Source: J Phys Chem B. 2024 Jul 9;128(28):6922–39. doi: 10.1021/acs.jpcb.4c01840 (PMC11264277; doi:10.1021/acs.jpcb.4c01840)
Supplement: Supplementary file 1 — jp4c01840_si_001.pdf [file jp4c01840_si_001.pdf]

Supporting Information for

Sub-Nanometer-Range Structural Effects From Mg^{2+} Incorporation in Na-Based Borosilicate Glasses Revealed by Heteronuclear NMR and MD Simulations

Peng Lv,[1,2,3] Baltzar Stevensson,[3] Renny Mathew,[3,4] Tieshan Wang,[1,2] and Mattias Edén[3,*]

[1]MOE Frontiers Science Center for Rare Isotopes, Lanzhou University, Lanzhou, 730000, PR China
[2]Key Laboratory of Special Function Materials and Structure Design Ministry of Education, Lanzhou University, Lanzhou, 730000, PR China
[3]Physical Chemistry Division, Department of Materials and Environmental Chemistry, Arrhenius Laboratory, Stockholm University, SE-106 91 Stockholm, Sweden
[4]Present address: Science Division, New York University Abu Dhabi, P.O. Box 129188, Abu Dhabi, United Arab Emirates

[*]Corresponding author. E-mail: *mattias.eden@mmk.su.se*

Contents

1. **Section S1**. Correlation Between $\overline{Z}_{\rm Na}$ and the Glass Composition.
2. **Section S2**. Relationship Between $M_2(F\text{–}F')$ and the Number of F–O–F' Linkages.
3. **Table S1**. Cation Field Strengths.
4. **Table S2**. MD Simulation Parameters.
5. **Table S3**. MD-Simulation Interatomic Potential Parameters.
6. **Table S4**. Dipolar Second Moments and Parameters Governing $M_2^{\rm rel}({\rm Si})$.
7. **Table S5**. Fractional Contribution of $M_2^{(2)}({\rm B}^{[p]}\text{–Si})$ to $M_2({\rm B}^{[p]}\text{–Si})$.
8. **Table S6**. Average F–O–F' Bond Lengths.
9. **Figure S1**. $\overline{Z}_{\rm Na} \sim n_{\rm O}/n_{\rm Na}$ Correlation Plot.
10. **References**.

S1 Correlation Between $\overline{Z}_{\mathrm{Na}}$ and the Glass Composition

Both average Na and Mg coordination numbers exhibit complex dependencies on the glass composition. All MgNaK–R glasses manifest a concurrent increase of $\overline{Z}_{\mathrm{Mg}}$ for decreasing K (Table **3**), i.e., for increasing $n_{\mathrm{B}}/n_{\mathrm{Si}}$ ratio, whereas any potential NBO dependence is unclear, where both MgNa4.0–0.75 and MgNa4.0–2.1 structures reveal essentially equal values $\overline{Z}_{\mathrm{Mg}} \approx 5.2$. The precise relationship between $\overline{Z}_{\mathrm{Na}}$ and the glass composition also appears to be complicated. Here we would like to clarify and revise some earlier statements made by Stevensson *et al.*:[S1] from a large ensemble of Na-bearing borate and boro(phospho)silicate glasses, we inferred that $\overline{Z}_{\mathrm{Na}}$ is increased for (*i*) decreasing NBO content, (*ii*) increasing B content (n_{B}), and for (*iii*) increasing $\mathrm{B}^{[4]}$ fraction ($x_{\mathrm{B}}^{[4]}$). However, while those inferences are born out for *some* glass series, several exceptions may be found. Hence, none of (*i*)–(*iii*) above holds generally and each has limited predictive power for $\overline{Z}_{\mathrm{Na}}$. One hurdle is that both the NBO content and the $\{x_{\mathrm{B}}^{[3]}, x_{\mathrm{B}}^{[4]}\}$ parameters are strongly correlated, but by non-linear relationships (eq **9**).

We therefore revisited our previously published $\{\overline{Z}_{\mathrm{Na}}\}$ data from Na and Ca/Na bearing borate, boro(phospho)silicate, and phosphosilicate glasses,[S1–S3] complemented by those presented herein. Fig. **S1** plots $\overline{Z}_{\mathrm{Na}}$ against the glass-stoichiometry ratios $n_{\mathrm{O}}/n_{\mathrm{Na}}$ and $n_{\mathrm{O}}/(n_{\mathrm{Na}} + n_M)$ for the Na and Ca/Na based glasses along with the Mg-bearing specimens of Table **1**. The results confirm the expectation that $\overline{Z}_{\mathrm{Na}}$ tends to increase for an increasing number of O atoms per Na^+ (or per M^{2+}) cation. However, while the $n_{\mathrm{O}}/n_{\mathrm{Na}}$ or $n_{\mathrm{O}}/(n_{\mathrm{Na}} + n_M)$ ratios offer a physically intuitive parameter for an approximate and qualitative assessment of the *relative* $\overline{Z}_{\mathrm{Na}}$ values of two BS glass stoichiometries, those molar ratios remain unreliable parameters, where several glasses clearly break the $\overline{Z}_{\mathrm{Na}} \sim n_{\mathrm{O}}/n_{\mathrm{Na}}$ or $\overline{Z}_{\mathrm{Na}} \sim n_{\mathrm{O}}/(n_{\mathrm{Na}}+n_M)$ relationship (Fig. **S1**). In particular, in the high NBO regime—i.e., for low $n_{\mathrm{O}}/n_{\mathrm{Na}}$ or $n_{\mathrm{O}}/(n_{\mathrm{Na}} + n_M)$ values—the data from the $\mathrm{Na_2O}$–$\mathrm{B_2O_3}$ and $\mathrm{Na_2O}$–CaO–$\mathrm{B_2O_3}$–$\mathrm{SiO_2}$–$\mathrm{P_2O_5}$ glass systems manifest a much steeper coordination change against $n_{\mathrm{O}}/n_{\mathrm{Na}}$ or $n_{\mathrm{O}}/(n_{\mathrm{Na}} + n_{\mathrm{Ca}})$ relative to their BS counterparts. Moreover, the coexistence of higher-CFS Ca^{2+} or Mg^{2+} cations in the glass tend to further smear out the $\overline{Z}_{\mathrm{Na}} \sim n_{\mathrm{O}}/(n_{\mathrm{Na}} + n_M)$ relationship.

Notably, some outlier data of Fig. **S1** appear to relate to trend (*ii*) above, i.e., that $\overline{Z}_{\mathrm{Na}}$ of a BS glass structure tends to increase for growing $n_{\mathrm{B}}/n_{\mathrm{Si}}$ ratio (i.e., decreasing K). That applies, for instance, to the four NaK–0.75 and MgNaK–0.75 glasses, for which $\{\overline{Z}_{\mathrm{Na}}\}$ adheres well with the dashed line of Fig. **S1** and revealing that the average coordination number is *decreased* by *either* the presence of Mg^{2+} cations *or* for increasing K (i.e., decreasing $n_{\mathrm{B}}/n_{\mathrm{Si}}$ ratio). The feature of an increased average Na coordination number in B-richer BS glasses mirrors previously reported higher $\overline{Z}_{\mathrm{Na}}$ and $\overline{Z}_{\mathrm{Ca}}$ values in aluminosilicate glasses relative to their silicate counterparts.[S4] However, the complex dependence of $\overline{Z}_{\mathrm{Na}}$ on the glass composition is (again) illustrated by the corresponding results for the NBO-rich NaK–2.1 and NaMgK–2.1 glasses, which manifests the *opposite* trends of *higher* $\overline{Z}_{\mathrm{Na}}$ values for the Mg-bearing glasses, while essentially equal average Na^+ coordination numbers are observed for both $K = \{2.0, 4.0\}$ structures.

Further work is required for elucidating the precise dependence of $\overline{Z}_{\mathrm{Na}}$ and $\overline{Z}_{\mathrm{Mg}}$ (or $\overline{Z}_M$ in general) on the oxide-based glass stoichiometry. Here, the presence of B complicates matters and it is likely that no simple and *general* relationship may be found.

S2 Relationship Between $M_2(F\text{–}F')$ and the Number of F–O–F' Linkages

The *targeted* information from the dipolar second-moment analysis is the assessment of the ratio between the average numbers $N(\text{B}^{[4]}\text{–O–Si})$ and $N(\text{B}^{[3]}\text{–O–Si})$ of Si atoms in the second coordination shells of the $\text{B}^{[4]}$ and $\text{B}^{[3]}$ sites, respectively, in the glass structure, i.e., the relative numbers of direct $\text{B}^{[4]}$–O–Si and $\text{B}^{[3]}$–O–Si linkages:

$$N^{\text{rel}} \equiv N(\text{B}^{[4]}\text{–O–Si})/N(\text{B}^{[3]}\text{–O–Si}). \tag{S1}$$

N^{rel} is approximated well (*vide infra*) by the corresponding dipolar second-moment ratio

$$M_2^{(2)\text{rel}}(\text{B}) \equiv M_2^{(2)}(\text{B}^{[4]}\text{–Si})/M_2^{(2)}(\text{B}^{[3]}\text{–Si}), \tag{S2}$$

where the superscript '(2)' of $M_2^{(2)}(\text{B}^{[p]}\text{–Si})$ implies that it was calculated from eq **6** when *solely* including the $r(\text{B}^{[p]}\text{–O–Si})$ distances to Si atoms in the *second* coordination sphere of $\text{B}^{[p]}$. Although $M_2^{(2)}(\text{B}^{[p]}\text{–Si})$ is readily obtained from the MD simulation-generated glass *model*, only the "fully converged" dipolar second moment, $M_2(\text{B}^{[4]}\text{–Si})$, is accessible by the $^{11}\text{B}\{^{29}\text{Si}\}$ REDOR NMR experiments. Therefore, in sections **3.5–3.7**, we only consider the NMR/MD-derived $M_2(\text{B}^{[4]}\text{–Si})$ and $M_2(\text{B}^{[3]}\text{–Si})$ data and their ratio

$$M_2^{\text{rel}}(\text{B}) \equiv M_2(\text{B}^{[4]}\text{–Si})/M_2(\text{B}^{[3]}\text{–Si}), \tag{S3}$$

along with the corresponding $\{M_2(\text{Si–B}^{[p]})\}$ sets (Table **5**).

Notably, although we consider the specific $M_2(\text{B}^{[p]}\text{–Si}) \sim N(\text{B}^{[p]}\text{–O–Si})$ correlation, all concepts and considerations herein apply to any $M_2(F\text{–}F') \sim N(F\text{–}F')$ relationship of F–O–F/F' linkages.[S5–S8]

S2.1 How Reliable is the $M_2^{\text{rel}}(\text{B}) \approx N^{\text{rel}}$ Approximation?

Here we examine the validity of the approximation

$$M_2^{\text{rel}}(\text{B}) \approx N^{\text{rel}}, \tag{S4}$$

which is primarily influenced by two assumptions/approximations: (I) The right side of eq S4 *solely* involve the relative numbers of *direct* $\text{B}^{[4]}/\text{B}^{[3]}$–O–Si linkages, whereas the left-side entity accounts *also* for all long-range $\text{B}^{[p]} \cdots \text{Si}$ distances. How well $M_2^{\text{rel}}(\text{B})$ matches N^{rel} depends directly on the approximation $M_2^{\text{rel}}(\text{B}) \approx M_2^{(2)\text{rel}}(\text{B})$. ($II$) The *identity* $M_2^{(2)\text{rel}}(\text{B}) = N^{\text{rel}}$ *only* holds strictly for identical sets of $\text{B}^{[3]} \cdots \text{Si}$ and $\text{B}^{[4]} \cdots \text{Si}$ distances in the structure. Below we assess the impact from each approximation (I) and (II), altogether suggesting that when N^{rel} is assessed *via* $M_2^{\text{rel}}(\text{B})$, both factors tend to *underestimate* the degree of $\text{B}^{[4]}$–O–Si bonding relative to $\text{B}^{[3]}$–O–Si, roughly by 20% and 4%, respectively. Altogether, that amounts to an ≈ 25 % underestimation of the degree of $\text{B}^{[4]}$–O–Si bonding when assessing N^{rel} from $M_2^{\text{rel}}(\text{B})$.

The approximation $M_2^{\text{rel}}(\text{B}) \approx M_2^{(2)\text{rel}}(\text{B})$ rests on the assumption that the shortest, and thereby most influential, $\text{B}^{[p]}$–O–Si distances underpinning $M_2^{(2)}(\text{B}^{[p]}\text{–Si})$ vastly dominate all other contributions to the converged experimental/calculated dipolar second moment, $M_2(\text{B}^{[p]}\text{–Si})$, in the glass structures. These values are related by

$$M_2^{(2)}(\text{B}^{[p]}\text{–Si}) = f^{(2)}(\text{B}^{[p]}\text{–Si})M_2(\text{B}^{[p]}\text{–Si}), \text{ with } p = \{3, 4\}. \tag{S5}$$

The MD-derived fractions $f^{(2)}(\mathrm{B}^{[p]}\text{–Si}) = M_2^{(2)}(\mathrm{B}^{[p]}\text{–Si})/M_2(\mathrm{B}^{[p]}\text{–Si})$ listed in Table **S5** only depend weakly on the precise glass composition. Yet $M_2^{(2)}(\mathrm{B}^{[4]}\text{–Si})$ constitutes a consistently larger portion of $M_2(\mathrm{B}^{[4]}\text{–Si})$, $f^{(2)}(\mathrm{B}^{[4]}\text{–Si}) \approx 0.84$ for the NBO-poor $R = 0.75$ glasses and $f^{(2)}(\mathrm{B}^{[4]}\text{–Si}) \approx 0.86$ for the NBO-rich $R = 2.1$ counterparts, relative to the overall lower but more confined values of $f^{(2)}(\mathrm{B}^{[3]}\text{–Si}) \approx 0.72 \pm 0.02$. Hence, as follows from the $f^{(2)}(\mathrm{B}^{[4]}\text{–Si})/f^{(2)}(\mathrm{B}^{[3]}\text{–Si})$ ratios presented in Table **S5**, the fully converged $M_2(\mathrm{B}^{[4]}\text{–Si})$ and $M_2(\mathrm{B}^{[3]}\text{–Si})$ estimates (eq S3) for approximating N^{rel} (eq S4) is bound to *underestimate* the number of $\mathrm{B}^{[4]}$–O–Si bridges relative to $\mathrm{B}^{[3]}$–O–Si by $\approx 20\%$.

We next consider the second factor that may potentially degrade the $M_2^{\mathrm{rel}}(\mathrm{B}) \approx N^{\mathrm{rel}}$ approximation, *as well as* breaking the much firmer $M_2^{(2)\mathrm{rel}}(\mathrm{B}) = N^{\mathrm{rel}}$ *identity*. The former is only strict when the sets of $\{\mathrm{B}^{[3]} \cdots \mathrm{Si}\}$ and $\{\mathrm{B}^{[4]} \cdots \mathrm{Si}\}$ distances are equal in the structure, which for a BS glass requires identical $r(\mathrm{B}^{[3]} \cdots \mathrm{Si})$ and $r(\mathrm{B}^{[4]} \cdots \mathrm{Si})$ distributions. However, to avoid dealing with an intractable problem, we only consider the *average* $\mathrm{B}^{[p]}$–O–Si bond lengths (Table **S6**), where the insurmountable hurdles associated with an accurate/exact handling of the bearings from parameter-distributions on *aggregate* experimental observables (such as $M_2(F\text{–}F')$ values) may be circumvented because they are expected to largely cancel across the distribution. Then, the $M_2^{(2)\mathrm{rel}}(\mathrm{B}) = N^{\mathrm{rel}}$ equality demands equal average $\mathrm{B}^{[4]}$–O–Si and $\mathrm{B}^{[3]}$–O–Si distances in the structure, whereas Table **S6** reveals that $\overline{r}(\mathrm{B}^{[4]}\text{–O–Si})$ is 2–4 pm longer than $\overline{r}(\mathrm{B}^{[3]}\text{–O–Si})$. Hence, the $[\overline{r}(F\text{–O–}F')]^{-6}$ dependence of the dipolar second moment $M_2(F\text{–}F')$ of eq **6** leads to a slight underestimation of the ratio between the numbers of $\mathrm{B}^{[4]}$–O–Si and $\mathrm{B}^{[3]}$–O–Si bonds when assessed from $M_2^{\mathrm{rel}}(\mathrm{B})$:

$$M_2^{\mathrm{rel}}(\mathrm{B}) \lesssim M_2^{(2)\mathrm{rel}}(\mathrm{B}) \approx N(\mathrm{B}^{[4]}\text{–O–Si})/N(\mathrm{B}^{[3]}\text{–O–Si}). \tag{S6}$$

Fortunately, the $\left[\overline{r}(\mathrm{B}^{[4]}\text{–O–Si})/\overline{r}(\mathrm{B}^{[3]}\text{–O–Si})\right]^{-6}$ ratios listed in Table **S4** suggest only marginal deviations from the $M_2^{(2)\mathrm{rel}}(\mathrm{B}) = N^{\mathrm{rel}}$ identity, where N^{rel} becomes underestimated by $\approx 4\%$ and $\approx 8\%$ for the BS glasses with $R = 0.75$ and $R = 2.1$, respectively. Hence, the (actual) number of $\mathrm{B}^{[4]}$–O–Si linkages (relative to $\mathrm{B}^{[3]}$–O–Si) is 4–8% higher than that suggested by the $M_2^{\mathrm{rel}}(\mathrm{B})$ values listed in Table **5**. Notably, one confirmation of the gross simplification of replacing distance-distributions by their averages is given by the good predictions of the $M_2^{\mathrm{rel}}(\mathrm{Si})$ values from *several* "aggregate" MD-derived structural parameters, encompassing the $\left[\overline{r}(\mathrm{B}^{[4]}\text{–O–Si})/\overline{r}(\mathrm{B}^{[3]}\text{–O–Si})\right]^{-6}$ factor based solely on the *average* $r(\mathrm{B}^{[p]}\text{–O–Si})$ distances (Table **S4** and section **3.7**).

We conclude that the experimental and/or modeled $M_2^{\mathrm{rel}}(\mathrm{B})$ ratio underestimates the degree of $\mathrm{B}^{[4]}$–O–Si bonding relative to $\mathrm{B}^{[3]}$–O–Si, meaning that the as-observed clear emphasis of $\mathrm{B}^{[4]}$–O–Si linkage-formation in the present glass structures nonetheless suggested by the $\{M_2^{\mathrm{rel}}(\mathrm{B})\}$ data (Table **S4** and section **3.5**) yet remains underestimated by $\approx 25\%$. We underscore that these considerations were enabled by having glass *models* available, whose predicted structures, however, are most likely to mimic those of the physical glasses well, as suggested by the very good agreement between the experimental and modeled $M_2^{\mathrm{rel}}(\mathrm{Si})$ results (Table **S4** and section **3.7**).

S2.2 Estimation of $M_2^{\mathrm{rel}}(\mathrm{stat})$

For a BS glass structure devoid of NBO anions and featuring a *strictly non-preferential* $\mathrm{B}^{[p]}$/Si intermixing, N^{rel} is given by the ratio of the two $\mathrm{B}^{[3]}$ and $\mathrm{B}^{[4]}$ coordination numbers, $N^{\mathrm{rel}}(\mathrm{stat}) = 4/3$. For that scenario, the $M_2^{\mathrm{rel}}(\mathrm{B})$ entity (eq S3) is denoted by $M_2^{\mathrm{rel}}(\mathrm{stat})$. However, Table **5** reveals distinctly different $P(F\text{–}F')$ preference-factors among the various network forming species, ranging from the strongest preference for $\mathrm{B}^{[3]}$–O–$\mathrm{B}^{[4]}$ linkage-formation to the weakest for $\mathrm{B}^{[4]}$–O–

$B^{[4]}$. Although neither of those $P(B^{[3]}–O–B^{[3]})$ and $P(B^{[4]}–O–B^{[4]})$ values nor the distinctly different $P(B^{[3]}–NBO) \approx 2.9$ and $P(B^{[4]}–NBO) \approx 0.5$ preference,[S9] *directly* affect the targeted selected sub-ensemble of $B^{[3]}/B^{[4]}$–O–Si linkages, they break the strict $N^{rel}(stat) = 4/3$ identity. Analysis of the glass models, however, revealed that these $B^{[4]}–O–B^{[3]}/B^{[4]}$ and $B^{[p]}$–NBO bonding effects largely cancel, yielding the average value of $N^{rel}(stat) = 1.44$ calculated across the NBO-poor glasses (Table **S5**), which is close to 4/3.

A representative $M_2^{rel}(stat)$ estimate may be derived from the $N^{rel}(stat) = 1.44$ value along with the correction factors $\left[\bar{r}(B^{[4]}–O–Si)/\bar{r}(B^{[3]}–O–Si)\right]^{-6}$ and $f^{(2)}(B^{[4]}–Si)/f^{(2)}(B^{[3]}–Si)$ that originate from the respective effects (*I*) and (*II*) of section **S2.1** (Table **S4**), yielding

$$M_2^{rel}(stat) \approx \frac{\left[\bar{r}(B^{[4]}–O–Si)/\bar{r}(B^{[3]}–O–Si)\right]^{-6} N^{rel}(stat)}{f^{(2)}(B^{[4]}–Si)/f^{(2)}(B^{[3]}–Si)} = \frac{0.96 \cdot 1.44}{1.167} = 1.18. \tag{S7}$$

The $M_2^{rel}(stat) \approx 1.18$ result applies to the Na*K*–0.75 and MgNa*K*–0.75 glasses *if* their $B^{[p]}$/Si intermixing is random/statistical (Table **S4**). We underscore that the prediction $M_2^{rel}(stat) \approx 1.18$ *only* holds *strictly* for BS glasses with (very) low NBO contents. In principle, $M_2^{rel}(stat)$ may be adjusted to encompass NBO-rich glasses by additionally accounting for the distinctly different preferences for $B^{[3]}$–NBO and $B^{[4]}$–NBO contacts, whose significant bearings progressively boost the number of Si–O–$B^{[4]}$/Si linkages in the glass network for increasing x_{NBO}; see section **3.5** and refs.[S9,S10] In the current absence of experimental data, however, such extensions require further resort to modeled bonding preference factors; e.g. see ref.[S9]

The accuracy of the $M_2^{rel}(stat) \approx 1.18$ result may be gauged from the MD-derived $M_2^{rel}(B)$ and $\{P(B^{[p]}–O–Si)\}$ data for each Na*K*–0.75 and MgNa*K*–0.75 glass model (Table **5**). The following equivalence holds identically for any modeled or physical glass structure,

$$M_2^{rel}(B)/M_2^{rel}(stat) = P(B^{[4]}–O–Si)/P(B^{[3]}–O–Si), \tag{S8}$$

and evaluates to unity for a statistical $B^{[p]}$/Si intermixing. Note that the entities on the left-hand side depends on the *number* of $B^{[p]}$–O–Si linkages in the glass, as opposed to the $P(B^{[4]}–O–Si)$ and $P(B^{[3]}–O–Si)$ factors, which represent the propensity for forming (*one*) $B^{[4]}$–O–Si and $B^{[3]}$–O–Si linkage, respectively. All of $M_2^{rel}(B)$, $P(B^{[4]}–O–Si)$, and $P(B^{[3]}–O–Si)$ are readily extracted from a given glass model with $R = 0.75$ (Table **5**), whereas $M_2^{rel}(stat)$ was *estimated* according to eq S7. Hence, all deviations between the left- and right-hand sides of eq S8 stem from errors in the $M_2^{rel}(stat) \approx 1.18$ approximation. It is gratifying that the discrepancies remain within $\approx 5\%$ for all Na*K*–0.75 and MgNa*K*–0.75 glass models, except for MgNa2.0–0.75 ($\approx 15\%$). Besides justifying eq. S7, these results underscore that the $M_2^{rel}(B)$ reduction observed both by MD simulations and NMR experiments for the Mg-bearing BS glasses originate from a reduced *preference* of $B^{[4]}$–O–Si bonding relative to $P(B^{[3]}–O–Si)$, i.e., a decreased ratio $P(B^{[4]}–O–Si)/P(B^{[3]}–O–Si)$, which is independent on the number of network linkages; see section **3.6**.

Table S1. Cation Field Strengths[a]

ion	CFS_M (Å^{-2})
$B^{[3]}$	1.60
$B^{[4]}$	1.39
$Si^{[4]}$	1.52
$Na^{[6]}$	0.18
$Ca^{[6]}$	0.36
$Mg^{[6]}$	0.46
$La^{[6]}$	0.52
$Y^{[6]}$	0.59
$Lu^{[6]}$	0.61
$Sc^{[6]}$	0.68

a Defined according to Dietzel,[S11] $\mathrm{CFS}_M = z/(r_M + r_\mathrm{O})^2$, where $r_\mathrm{O} = 1.36$ Å and r_M is the cation radius,[S12] where $M^{[6]}$ coordinations were assumed for all M^+, M^{2+}, and M^{3+} glass-network *modifiers.*

Table S2. MD Simulation Parameters[a]

glass	oxide equivalents (mol%)				number of atoms								
	MgO	Na_2O	B_2O_3	SiO_2	N_{tot}	N_{Mg}	N_{Na}	N_B	N_{Si}	N_O	N_{sim}	a (nm)[b]	ρ (g cm^{-3})[c]
Na2.0–0.75		0.200	0.267	0.533	6784		768	1024	1024	3968	4	4.342	2.454
MgNa2.0–0.75	0.100	0.100	0.267	0.533	6592	192	384	1024	1024	3968	4	4.361	2.339
Na4.0–0.75		0.130	0.174	0.696	9856		768	1024	2048	6016	4	5.022	2.393
MgNa4.0–0.75	0.065	0.065	0.174	0.696	9664	192	384	1024	2048	6016	4	5.069	2.274
Na2.0–2.1		0.412	0.196	0.392	8650		2100	1000	1000	4550	4	4.744	2.488
MgNa2.0–2.1	0.206	0.206	0.196	0.392	8125	525	1050	1000	1000	4550	4	4.630	2.486
Na4.0–2.1		0.296	0.141	0.563	11650		2100	1000	2000	6550	4	5.270	2.496
MgNa4.0–2.1	0.148	0.148	0.141	0.563	11125	525	1050	1000	2000	6550	4	5.209	2.451

[a] Parameters employed in the MD simulations, where $\{N_{Mg}, N_{Na}, N_B, N_{Si}, N_O\}$ denote the number of {Mg, Na, B, Si, O} atoms out of the total number $N_{tot} = N_{Mg} + N_{Na} + N_B + N_{Si} + N_O$ in the model, while N_{sim} represents the number of independent glass models generated (section **2.3**).

[b] Side length of the periodic cubic box.

[c] Experimental glass density (uncertainty ± 0.004 g cm^{-3}) reproduced from Lv *et al.*[S13]

Table S3. MD-Simulation Interatomic Potential Parameters[a]

atom-pair α–β	atom-pair potential parameters $A_{\alpha\beta}$ (keV)	$\rho_{\alpha\beta}$ (Å)	$C_{\alpha\beta}$ (eVÅ^6)	$D_{\alpha\beta}$ (eVÅ^{12})	ref.
Na–O_S	56.465	0.1939	0	0	S14
Ca–O_S	2.152	0.3092	0.099	0	S14
Mg–O_S	3.886	0.2533	0	0	S15
B–O_S	0.472	0.3350	0	5.4	S16[b]
Si–O_S	1.284	0.3205	10.662	0	S17
O_S–O_S	22.764	0.1490	27.880	0	S17
O_C–O_S	0	0	0	0.1	S18
α–β–α	three-atom potential parameters[c] $k_{\alpha\beta\alpha}$ (eV/rad^2)	$\rho_{\alpha\beta\alpha}$ (Å)	$\theta^0_{\alpha\beta\alpha}$		
O_S–Si–O_S	5.48	2.030	109.47°		S18

[a] Interatomic pair-potential parameters of the modified Buckingham potential of eq **5**.

[b] B–O force-field parameters were first utilized in a report on borophosphosilicate glass structures.[S16] Their derivation along with further validations were given by Stevensson *et al.*[S1]

[c] Three-atom potential parameters, represented by a truncated harmonic function,[S18] $U_{\alpha\beta\alpha} = \frac{1}{2}k_{\alpha\beta\alpha}\left(\theta_{ijk} - \theta^0_{\alpha\beta\alpha}\right)^2 \exp\left\{-(r^8_{ij} + r^8_{jk})/\rho^8_{\alpha\beta\alpha}\right\}$, constitute a modified functional of that reported by Tilocca *et al.*[S19] They were evaluated out to r =250 pm.[S20]

Table S4. Dipolar Second Moments and Parameters Governing M_2^{rel}(Si) [a]

glass	$M_2(\mathrm{B}^{[3]}$–Si) (10^4 Hz^2)	$M_2(\mathrm{B}^{[4]}$–Si) (10^4 Hz^2)	M_2^{rel}(B) [b]	$M_2^{(2)\mathrm{rel}}$(B) [b]	M_2(Si–$\mathrm{B}^{[3]}$) (10^4 Hz^2)	M_2(Si–$\mathrm{B}^{[4]}$) (10^4 Hz^2)	M_2^{rel}(Si) [c]	$M_2^{(2)\mathrm{rel}}$(Si) [c]	Π [d]	$x_{\mathrm{B}}^{[4]}/x_{\mathrm{B}}^{[3]}$	P^{rel}(Si) [e]	$\bar{r}^{\mathrm{rel}}$ [f]
Na2.0–0.75	5.13	7.58	1.48	1.78	9.32	16.61	1.78	2.17	2.13	1.21	1.28	0.96
MgNa2.0–0.75	5.41	7.16	1.32	0.72	14.02	10.14	0.72	0.83	0.80	0.55	1.04	0.97
Na4.0–0.75	7.08	9.51	1.34	1.50	6.70	10.04	1.50	1.77	1.78	1.12	1.16	0.95
	3.92	5.65	1.44	1.50	3.00	6.99	2.33	1.77	2.58	1.62	1.16	0.95
MgNa4.0–0.75	7.23	8.85	1.22	0.53	10.12	5.33	0.53	0.59	0.58	0.43	0.98	0.96
	3.84	4.02	1.05	0.53	5.30	2.50	0.47	0.59	0.61	0.45	0.98	0.96
Na2.0–2.1	3.47	6.72	1.94	1.57	7.68	12.04	1.57	1.90	1.30	0.81	1.20	0.93
MgNa2.0–2.1	3.78	6.51	1.72	0.99	9.63	9.49	0.99	1.20	0.84	0.57	1.12	0.92
Na4.0–2.1	5.14	8.84	1.72	2.66	4.04	10.75	2.66	3.25	2.51	1.55	1.21	0.93
MgNa4.0–2.1	5.55	8.45	1.53	1.22	6.18	7.51	1.22	1.46	1.15	0.80	1.09	0.92
	3.37	5.11	1.51	1.22	3.75	4.55	1.21	1.46	1.17	0.80	1.09	0.92
σ [g]	0.04	0.05	0.02	0.04	0.10	0.12	0.04	0.05	0.04	0.02	0.02	0.01

[a] Dipolar second moments obtained either by MD simulations or NMR experiments (the latter data appear on the line below the glass label).

[b] Ratio of NMR or MD derived dipolar second moments defined by eqs S3 and S2.

[c] Defined analogously with eqs S3 and S2: $M_2^{\mathrm{rel}}(\mathrm{Si}) = M_2(\mathrm{Si–B}^{[4]})/M_2(\mathrm{Si–B}^{[3]})$ and $M_2^{(2)\mathrm{rel}}(\mathrm{Si}) = M_2^{(2)}(\mathrm{Si–B}^{[4]})/M_2^{(2)}(\mathrm{Si–B}^{[3]})$.

[d] Product of the factors listed in the subsequent columns, $\Pi = N^{\mathrm{rel}}(\mathrm{stat})(x_{\mathrm{B}}^{[4]}/x_{\mathrm{B}}^{[3]})P^{\mathrm{rel}}(\mathrm{Si})\bar{r}^{\mathrm{rel}}$, which approximates M_2^{rel}(Si) with $N^{\mathrm{rel}}(\mathrm{stat}) = 1.44$ (section **S2**).

[e] $P^{\mathrm{rel}}(\mathrm{Si}) \equiv P(\mathrm{Si–O–B}^{[4]})/P(\mathrm{Si–O–B}^{[3]}) = P(\mathrm{B}^{[4]}\mathrm{–O–Si})/P(\mathrm{B}^{[3]}\mathrm{–O–Si})$, with the preference-factor values listed in Table **5**.

[f] Ratio $\bar{r}^{\mathrm{rel}} = [\bar{r}(\mathrm{Si–O–B}^{[4]})/\bar{r}(\mathrm{Si–O–B}^{[3]})]^{-6}$ with the $\bar{r}(\mathrm{Si–O–B}^{[p]})$ distances given in Table **S6**.

[g] The data uncertainties are $\pm 1\sigma$ with σ given for each entity.

Table S5. Fractional Contribution of $M_2^{(2)}$(B$^{[p]}$–Si) to M_2(B$^{[p]}$–Si)

glass	$f^{(2)}$(B$^{[3]}$–Si) [a]	$f^{(2)}$(B$^{[4]}$–Si) [a]	$\frac{f^{(2)}(\text{B}^{[4]}\text{–Si})}{f^{(2)}(\text{B}^{[3]}\text{–Si})}$ [b]	N^{rel}(stat) [c]	M_2^{rel}(stat) [d]	M_2^{rel}(B)/M_2^{rel}(stat)
Na2.0–0.75	0.696	0.848	1.22	1.44	1.19	1.24
MgNa2.0–0.75	0.727	0.839	1.15	1.48	1.22	1.09
Na4.0–0.75	0.717	0.844	1.18	1.41	1.16	1.15
MgNa4.0–0.75	0.748	0.837	1.12	1.44	1.19	1.03
Na2.0–2.1	0.727	0.881	1.21	2.08	1.71	1.13
MgNa2.0–2.1	0.710	0.862	1.21	2.01	1.65	1.04
Na4.0–2.1	0.705	0.862	1.22	1.84	1.52	1.13
MgNa4.0–2.1	0.705	0.848	1.20	1.79	1.48	1.03
σ [e]	0.003	0.002	0.01	0.01	0.01	0.02

[a] $f^{(2)}$(B$^{[p]}$–Si) = $M_2^{(2)}$(B$^{[p]}$–Si)/M_2(B$^{[p]}$–Si) calculated from eq S5, where $M_2^{(2)}$(B$^{[p]}$–Si) and M_2(B$^{[p]}$–Si) were obtained from eq **6** by accounting *solely* for the *direct* B$^{[p]}$–O–Si distances and *all* B$^{[p]}$–O–Si distances, respectively, in the MD-derived glass model. Note that $f^{(2)}$(B$^{[p]}$–Si) $\equiv$ $f^{(2)}$(Si–B$^{[p]}$).

[b] Defined by eq S5.

[c] N^{rel}(stat) calculated from the glass models and representing the as-observed number of bonds at the {B$^{[4]}$} sites relative to those of {B$^{[3]}$} that are accessible for B$^{[p]}$–O–Si bonding, i.e., after excluding all B$^{[p]}$–O–B$^{[q]}$ linkages and B$^{[p]}$–NBO bonds.

[d] M_2^{rel}(stat) calculated from eq S7 but using the as-stated MD-derived N^{rel}(stat) value of each glass model.

[e] The data uncertainties are $\pm 1\sigma$ with σ given for each entity.

Table S6. Average F–O–F' Bond Lengths[a]

glass	$\bar{r}$(Si–O–F) (pm)			$\bar{r}$($B^{[3]}$–O–F) (pm)		$\bar{r}$($B^{[4]}$–O–F) (pm)
	Si	$B^{[3]}$	$B^{[4]}$	$B^{[3]}$	$B^{[4]}$	$B^{[4]}$
Na2.0–0.75	310.2	276.1	278.2	244.9	252.2	252.5
MgNa2.0–0.75	310.3	275.9	277.3	243.7	252.6	253.3
Na4.0–0.75	309.7	275.3	277.6	242.7	252.0	252.6
MgNa4.0–0.75	309.9	275.4	277.4	243.0	252.2	252.7
Na2.0–2.1	310.9	277.3	280.6	247.4	257.0	258.2
MgNa2.0–2.1	310.3	276.2	280.1	246.7	256.7	259.2
Na4.0–2.1	309.8	276.1	279.3	244.4	254.3	255.6
MgNa4.0–2.1	309.9	275.6	279.2	245.1	255.7	256.7
σ [b]	0.1	0.1	0.1	0.5	0.5	1.1

[a] MD-derived average bond lengths, $\bar{r}(F$–O–$F')$, for the network formers $\{F, F'\} = \{$Si, $B^{[3]}$, $B^{[4]}\}$.

[b] The data uncertainties are $\pm 1\sigma$ with σ given for each entity.

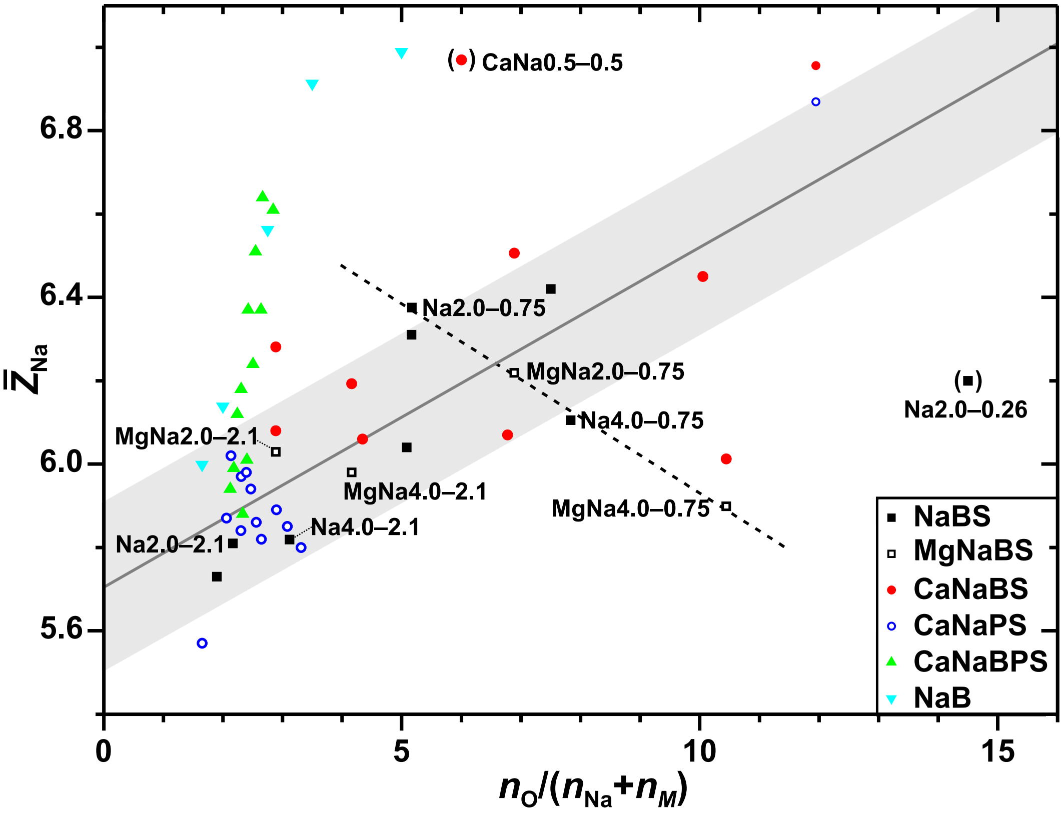

Fig. S1. MD-derived average coordination numbers of Na ($\overline{Z}_{\text{Na}}$) plotted against either the $n_{\text{O}}/n_{\text{Na}}$ molar ratio for Na_2O–B_2O_3–(SiO_2) glasses, or $n_{\text{O}}/(n_{\text{Na}} + n_M)$ for CaO–Na_2O–(B_2O_3)–SiO_2–P_2O_5 and CaO/MgO–Na_2O–B_2O_3–SiO_2 glasses. The data from the present NaK–R and MgNaK–R glasses (Table **1**) are marked, where the NaK–0.75 and MgNaK–0.75 results follow the linear trend marked by the dashed black line, implying a linear decrease of $\overline{Z}_{\text{Na}}$ for increasing K or by partially replacing Na_2O by MgO. All other $\{\overline{Z}_{\text{Na}}\}$ data are reproduced from refs.[S1,S3] The grey line marks the result from fitting $\overline{Z}_{\text{Na}}$ against $n_{\text{O}}/(n_{\text{Na}} + n_M)$ for all BS glasses incorporating Na^+, Ca^{2+}, and/or Mg^{2+} along with their phosphosilicate counterparts ("CaNaPS"), but excluding all borate ("NaB") and CaNa-based borophosphosilicate ("CaNaBPS") glasses and a few other outlier data points enclosed by parentheses. That yielded a very modest correlation coefficient of $R^2 = 0.56$. The grey area around the best-fit line serves to guide the eye to appreciate the spread of the data upon which the fit was based.

References

(S1) Stevensson, B.; Yu, Y.; Edén, M. Structure–Composition Trends in Multicomponent Borosilicate-Based Glasses Deduced from Molecular Dynamics Simulations with Improved B–O and P–O Force Fields. *Phys. Chem. Chem. Phys.* **2018**, *20*, 8192–8209.

(S2) Mathew, R.; Stevensson, B.; Edén, M. Na/Ca Intermixing around Silicate and Phosphate Groups in Bioactive Phosphosilicate Glasses Revealed by Heteronuclear Solid-State NMR and Molecular Dynamics Simulations. *J. Phys. Chem. B* **2015**, *119*, 5701–5715.

(S3) Yu, Y.; Stevensson, B.; Edén, M. A Unified ^{23}Na NMR Chemical Shift Correlation with Structural Parameters in Multicomponent Silicate-Based Glasses. *J. Am. Ceram. Soc.* **2020**, *103*, 762–767.

(S4) Cormier, L.; Neuville, D. R. Ca and Na Environments in Na_2O–CaO–Al_2O_3–SiO_2 Glasses: Influence of Cation Mixing and Cation-Network Interactions. *Chem. Geol.* **2004**, *213*, 103–113.

(S5) Zhang, L.; Eckert, H. Short- and Medium-Range Order in Sodium Aluminophosphate Glasses: New Insights from High-Resolution Dipolar Solid-State NMR Spectroscopy. *J. Phys. Chem. B* **2006**, *110*, 8946–8958.

(S6) Eckert, H. Advanced Dipolar Solid State NMR Spectroscopy of Glasses, in *Modern Glass Characterization*, M. Affatigato Ed., John Wiley & Sons, Ltd. **2015**, *9*, 1–46.

(S7) Eckert, H. Spying with Spins on Messy Materials: 60 Years of Glass Structure Elucidation by NMR Spectroscopy. *Int. J. Appl. Glass Sci.* **2017**, *9*, 167–187.

(S8) Edén, M. NMR of Glasses, in *NMR of Inorganic Nuclei*, Ed. Bryce, D. L., in *Comprehensive Inorganic Chemistry III*, Eds. Reedijk, J., Poeppelmeier, K. R, Oxford. Elsevier. **2023**, *9*, 583–659.

(S9) Lv, P.; Stevensson, B.; Yu, Y.; Wang, T.; Edén, M. BO_3/BO_4 Intermixing in Borosilicate Glass Networks Probed by Double-Quantum ^{11}B NMR: What Factors Govern BO_4–BO_4 Formation?. *J. Phys. Chem. C* **2023**, *127*, 20026–20040.

(S10) Yu, Y.; Stevensson, B.; Edén, M. Direct Experimental Evidence for Abundant BO_4–BO_4 Motifs in Borosilicate Glasses from Double-Quantum ^{11}B NMR Spectroscopy. *J. Phys. Chem. Lett.* **2018**, *9*, 6372–6376.

(S11) Dietzel, A. Die Kationenfeldstärken und ihre Beziehungen zu Entglasungsvorgängen, zur Verbindungsbildung und zu den Schmelzpunkten von Silicaten. *Z. Electrochem.* **1942**, *48*, 9–23.

(S12) Shannon, R. D. Revised Effective Ionic-Radii and Systematic Studies of Interatomic Distances in Halides and Chalcogenides. *Acta Cryst.* **1976**, *A32*, 751–767.

(S13) Lv, P.; Wang, C.; Stevensson, B.; Yu, Y.; Wang, T.-S.; Edén, M. Impact of the Cation Field Strength on Physical Properties and Structures of Alkali and Alkaline-Earth Borosilicate Glasses. *Ceram. Int.* **2022**, *48*, 18094–18107.

(S14) Tilocca, A.; de Leeuw, N. H.; Cormack, A. N. Shell-Model Molecular Dynamics Calculations of Modified Silicate Glasses. *Phys. Rev. B.* **2006**, *73*, 104209.

(S15) Pedone, A.; Malavasi, G.; Menziani, M. Computational Insight into the Effect of CaO/MgO Substitution on the Structural Properties of Phospho-Silicate Bioactive Glasses. *J. Phys. Chem. C* **2009**, *113*, 15723–15730.

(S16) Yu, Y.; Stevensson, B.; Edén, M. Medium-Range Structural Organization of Phosphorus-Bearing Borosilicate Glasses Revealed by Advanced Solid-State NMR Experiments and MD Simulations: Consequences of B/Si Substitutions. *J. Phys. Chem. B* **2017**, *121*, 9737–9752.

(S17) Sanders, M. J.; Leslie, M.; Catlow, C. R. A. Interatomic Potentials for SiO_2. *J. Chem. Soc., Chem. Commun.* **1984**, , pp. 1271–1273.

(S18) Mathew, R.; Stevensson, B.; Tilocca, A.; Edén, M. Toward a Rational Design of Bioactive Glasses with Optimal Structural Features: Composition-Structure Correlations Unveiled by Solid-State NMR and MD Simulations. *J. Phys. Chem. B* **2014**, *118*, 833–844.

(S19) Tilocca, A.; Cormack, A. N.; de Leeuw, N. H. The Structure of Bioactive Silicate Glasses: New Insights from Molecular Dynamics Simulations. *Chem. Mater.* **2007**, *19*, 95–103.

(S20) Todorov, I. T.; Smith, W.; Trachenko, K.; Dove, M. T. DL_POLY_3: New Dimensions in Molecular Dynamics Simulations via Massive Parallelism. *J. Mater. Chem.* **2006**, *16*, 1911–1918.
